# A comparison of the beta‐geometric model with landmarking for dynamic prediction of time to pregnancy

**DOI:** 10.1002/bimj.201900155

**Published:** 2019-11-18

**Authors:** Rik van Eekelen, Hein Putter, David J. McLernon, Marinus J. Eijkemans, Nan van Geloven

**Affiliations:** ^1^ Centre for Reproductive Medicine, Amsterdam UMC, Academic Medical Centre University of Amsterdam Amsterdam The Netherlands; ^2^ Medical Statistics, Department of Biomedical Data Sciences Leiden University Medical Centre Leiden The Netherlands; ^3^ Medical Statistics Team Institute of Applied Health Sciences University of Aberdeen Aberdeen UK; ^4^ Department of Biostatistics and Research Support, Julius Centre University Medical Centre Utrecht Utrecht The Netherlands

**Keywords:** beta‐geometric model, Cox model, dynamic prediction, frailty, heterogeneity, landmarking, time to pregnancy

## Abstract

We conducted a simulation study to compare two methods that have been recently used in clinical literature for the dynamic prediction of time to pregnancy. The first is landmarking, a semi‐parametric method where predictions are updated as time progresses using the patient subset still at risk at that time point. The second is the beta‐geometric model that updates predictions over time from a parametric model estimated on all data and is specific to applications with a discrete time to event outcome. The beta‐geometric model introduces unobserved heterogeneity by modelling the chance of an event per discrete time unit according to a beta distribution. Due to selection of patients with lower chances as time progresses, the predicted probability of an event decreases over time. Both methods were recently used to develop models predicting the chance to conceive naturally. The advantages, disadvantages and accuracy of these two methods are unknown. We simulated time‐to‐pregnancy data according to different scenarios. We then compared the two methods by the following out‐of‐sample metrics: bias and root mean squared error in the average prediction, root mean squared error in individual predictions, Brier score and c statistic. We consider different scenarios including data‐generating mechanisms for which the models are misspecified. We applied the two methods on a clinical dataset comprising 4999 couples. Finally, we discuss the pros and cons of the two methods based on our results and present recommendations for use of either of the methods in different settings and (effective) sample sizes.

## 1 INTRODUCTION

Clinical prediction models can be utilized by clinicians and patients to inform medical decision making in a shared, personalized and evidence‐based manner (Moons, Royston, Vergouwe, Grobbee, & Altman, 2009). Typical examples are to predict the probability that a disease is present to justify the use of an invasive or expensive diagnostic test, or prediction of the expected course of a disease to aid in deciding whether treatment should be initiated. However, there is rarely solely one fixed moment in time when a medical decision has to be made. More often, clinicians make an initial decision based on information known at baseline, but wish to reassess this decision later on, since during the period between assessments often additional or updated information has become available (Schumacher, Hieke, Ihorst, & Engelhardt, 2019). Examples include (re)measured biomarkers or an intermediate event that the patient can experience. The fact that the patient has remained event‐free until the time of reassessment is also important information that can be used to update the patient's prognosis (van Houwelingen & Putter, 2012).

Statistical methods for dynamic prediction have been introduced that allow predictions to be updated over time. This enables the clinicians and patients to make an informed decision at later time points based on the most recent information. Dynamic prediction thus has a high clinical utility (Schumacher et al., 2019). Dynamic prediction models are being used more often in recent years as shown by 86 search results for ‘dynamic prediction’ on PubMed in 2000 and 603 results in 2017. Further, several published models in oncology and cardiology are ready for clinical use via a web‐based calculator or nomogram (Eichinger, Heinze, & Kyrle, 2014; Fontein et al., 2015).

One setting where there is an explicit need for dynamic prediction is in reproductive medicine, in particular the subspecialisation of fertility. Approximately 1 in 10 heterosexual couples who wish to have a child cannot conceive naturally within one year of trying and this is referred to as subfertility (Gnoth et al., 2005). Subfertile couples receive a fertility workup wherein the basic necessities to achieve a natural pregnancy are established, but in 40–50% no barrier can be found, which is referred to as unexplained subfertility (Aboulghar et al., 2009). Treatment (medically assisted reproduction, MAR) is available, for example, intrauterine insemination or in vitro fertilisation, but since couples without a clear‐cut diagnosis can still conceive naturally, the preferred treatment course is not evident (van Eekelen et al., 2017b).

To make decisions on whom to treat, clinicians may turn to statistical models to inform them on the chance of pregnancy when following alternative scenarios. For instance, if a couple's prognosis of natural conception is relatively good, they might not need invasive MAR treatment but could instead keep trying to conceive naturally. If their prognosis is poor, the couple is expected to benefit from MAR and they can be treated. The initial decision is made upon completion of the diagnostic workup. However, if a couple choose to continue trying to conceive naturally for an additional period of time and do no succeed, again a decision has to be made when the couple return to the clinic.

‘Static’ models that predict over one fixed time horizon are unable to update predictions and will overestimate the probability of conception when reapplied at a later time (van Eekelen et al., 2017b). This is because the prognosis of natural conception decreases as time progresses due to a selection process that occurs. This is caused by couples who have a high chance of natural conception conceiving earlier and dropping out of the population, leaving behind a subset that, on average, has a lower probability of conception (van Eekelen et al., 2017b). Several statistical methods and models have been suggested to estimate the prognosis of (natural) conception in the presence of heterogeneity, but most focus on retrospective or cross‐sectional studies regarding time‐to‐pregnancy since those are the most cost‐efficient study designs (Ecochard, 2006; Scheike & Jensen, 1997; Scheike & Keiding, 2006; Zhou, 2006). These methods do not naturally extend to a setting of dynamic prediction.

Recently, two dynamic prediction models have been developed in the setting of reproductive medicine, published in a high impact clinical journal in the field (McLernon et al., 2019; van Eekelen et al., 2017a). The statistical methods used in these two publications are quite different: one used the landmarking in combination with Cox proportional hazards models and the other used the beta‐geometric model (Bongaarts, 1975; van Houwelingen & Putter, 2012; Weinberg & Gladen, 1986).

It is unknown what the advantages or disadvantages are of these two methods and how their predictive accuracy compares.

The aim of this paper is to identify which of these two methods perform best when used to develop a clinical dynamic prediction model. This cannot formally be assessed in an internal validation of the developed prediction models as the truth that they are estimating is unknown. We conducted a simulation study based on the setting of predicting the chances of pregnancy to compare these methods and to advise which method should be preferred in a particular setting. We applied the two methods on a clinical data set comprising 4999 couples with unexplained subfertility.

## 2 METHOD DESCRIPTION

### 2.1 Landmarking with Cox proportional hazard models

The first method to derive dynamic predictions of time to pregnancy is landmarking. This method was recently used by McLernon et al. (2019) to develop a clinical prediction model for time to pregnancy. Landmarking was first suggested by Anderson, Cain, and Gelber (1983) to prevent immortal time bias induced in survival modelling in oncology. In this original proposal, the authors suggested creating new datasets, the landmark datasets, at fixed time points after inclusion in the study using only patients who have survived at least until that time point. Covariates are updated based on information that has become available since the start of follow‐up, for example, a surgery that was since performed, after which the effect of those covariates can be estimated in the landmarks in the absence of immortal time bias.

Landmarking for dynamic prediction was introduced by van Houwelingen (2007). We define *s* as the landmark time point, *w* as the fixed prediction window, that is the time span that each landmark covers and *t* as the follow‐up time after inclusion in the study. The prediction window can be any time period over which one wants to predict, it is not related to the distance between landmarks. Van Houwelingen (2007) suggests to select a set of *K* + 1 landmark time points {*s*_0_, *s*_1_ *, s_j_…, s_K_*} and then to create a landmark dataset for each landmark time point by only selecting patients from the original dataset that are still at risk at *s_j_* and censoring them at time *s_j_ + w* (often referred to as administrative censoring).

After creating these landmark datasets, there are three approaches to estimate the dynamic predictions over time. The first and easiest option is to fit a separate Cox model on every landmark dataset and use these separate models to derive dynamic predictions. We get *K* + 1 Cox models:

(1) $$S_{ij}\;\left(s_{j}+w|X_{ij}\right)=S_{0j}{\left(s_{j}+w\right)}^{\exp(\sum \gamma_{j}X_{ij})}$$

with

$$S_{0j}\;\left(s_{j}+w\right)=\;\exp\left(-H_{0j}\left(s_{j}+w\right)\right),$$

where $S_{ij}\left(s_{j}+w|X_{ij}\right)$ is the probability that subject *i* with covariate values *X_ij_* at landmark time point *s_j_* will survive until at least *s_j_+w* and $\gamma_{j}$ are the estimated regression coefficients for covariates *X*. We assume that the $\gamma_{j}$ are constant over time, that is the assumption of proportional hazards is met, at least within landmarks. The cumulative hazard *H*_0_ *_j_* is estimated based on the follow‐up information of subjects at risk from *s_j_*.

Using separate Cox models in landmarking has the advantage of being semi‐parametric and thus not reliant on the shape of the underlying distribution of the baseline cumulative hazard. The method can easily incorporate time‐varying covariates; however, it might be sensitive to small sample sizes in later landmark datasets when estimating separate baseline hazards and separate regression coefficients based on limited sample sizes.

If it is not expected that effects of covariates will change over time, or if that does not align with the research question, one might wish to fit a simpler, more concise model. Van Houwelingen and Putter (2012) propose a second approach referred to as the ipl (integrated partial likelihood) ‘super’ model (van Houwelingen & Putter, 2012). The process is equivalent to the first approach in that it uses the constructed landmark datasets at time points *s_j_*, but instead of analysing them separately, we now assume that there is only one fixed set of coefficients γ. Thus, we assume that all γ*_j_* from (1) are equal to γ and that not only do they remain constant over time within a landmark, but also across landmarks. γ is estimated using likelihood contributions from all landmark sets, which might improve the stability of the estimations. In this second approach, the baseline hazards are still estimated separately for each landmark. In practice, the parameters for this second approach can be estimated by using landmark as a ‘strata’ variable when estimating one Cox model on a ‘stacked’ dataset consisting of all landmark datasets.

The third approach, referred to as the ipl* super model, is to simplify the model further by assuming that the baseline hazards across landmarks are related. One can assume a functional form of the baseline hazard by adding (functions of) *s_j_* to the model. We get

(2) $$S_{0j}\left(s_{j}+w\;|\;s_{j}\right)=\exp(-H_{0}\left(w\right)\;\exp\left(\Gamma (s_{j})\right),$$

where $\exp(\Gamma (s_{j}))$ is the assumed fixed ratio between the cumulative hazard at landmark *s_j_* relative to the cumulative hazard at *s*_0_. This function can comprise multiple parameters that are usually incorporated using linear and quadratic terms for transformed values of *s_j_* in landmarks, dividing by *s_K_* such that the range of values for the terms becomes 0 to 1(van Houwelingen & Putter, 2012). Alternatively, we can also formulate the ipl* super model by rearranging (2), yielding an expression where $\Gamma (s_{j})$ is modelled as additional coefficients in the regression part of the Cox model,

(3) $$S_{ij}\left(s_{j}+w\;|\;s_{j},X_{ij}\right)=S_{0}{\left(w\right)}^{\exp\left(\sum \gamma X_{ij}+\Gamma \left(s_{j}\right)\right)}$$

with

$$S_{0}\;\left(w\right)=\exp\left(-H_{0}\left(w\right)\right).$$

The advantage of this third ipl* approach is that it avoids estimation of many separate baseline hazards by pooling the baseline hazard of all landmarks at the cost of assuming a functional form of how the baseline hazard changes over landmarks. An additional advantage is that the ipl* model no longer depends on the choice of values for *s_j_*: The model can predict over time horizons exceeding *s_j_+w* or at time points not included in *s_j_*, which is something the ipl model cannot do since there are no estimated baseline hazards between landmarks or after the prediction window.

When applying landmarking in combination with Cox models in the fertility setting, the separate baseline hazards will accommodate the decrease in chances over time for the ‘separate Cox models’ and the ipl super model. For the ipl* super model, the decrease in chances is captured by the function $\Gamma (s_{j})$.

### 2.2 Beta‐geometric model

The second method to derive dynamic predictions from time‐to‐pregnancy data is the parametric beta‐geometric model introduced by Bongaarts (1975). This method was recently used by van Eekelen et al. (2017a) to develop a clinical prediction model for time to pregnancy. The beta‐geometric model is based on the notion that given a per cycle probability to conceive *p* that holds for all couples, time to conception *T* will follow a geometric distribution (Bongaarts, 1975). In the context of conception and subfertility, the cycle refers to every menstrual cycle, that is every opportunity for a couple to conceive. The probability of a first pregnancy in cycle *t* is thus

(4) $$P\left(T=t\;|\;p\right)=p{\left(1-p\right)}^{t-1}.$$

However, *p* is unlikely to be equal for all individual couples who wish to conceive and the spread in chances is modelled according to a beta distribution (Bongaarts, 1975; Leridon & Spira, 1984; te Velde, Eijkemans, & Habbema, 2000). The two shape parameters of the *beta* distribution, *α* and *β*, are reparametrized following Griffiths (1973) to

(5) $$\mu =\frac{\alpha }{\alpha +\beta },\;\theta =\frac{1}{\alpha +\beta },$$

where *μ* is the mean probability of conception in the first cycle and *θ* is now the second shape parameter that represents the extent of heterogeneity (Griffiths, 1973; Weinberg & Gladen, 1986). After *s* failed cycles, there remains a subset of couples with on average a lower probability per cycle *p*, such that *p* will follow a beta distribution with updated *μ* and *θ*

(6) $$\mu_{s}=\frac{\mu }{1+s\theta }\;\mathrm{and}\;\theta_{s}=\frac{\theta }{1+s\theta }.$$

The probability to conceive within *s + w* cycles given that a couple did not conceive within *s* cycles is

(7) $$P\left(T\leq s+w\;|\;T〉s\right)=1-\prod_{j=s}^{s+w-1}\frac{1-\mu +j\theta }{1+j\theta }.$$

To incorporate baseline covariate information *X*, we follow Weinberg and Gladen (1986) and regress the logit of the population mean *μ* linearly on the covariates *X*. We use a log link for *θ* and get

(8) $$\mu \left(X_{i}\right)=\frac{\exp\left(\mu_{0}+\sum \beta X_{i}\right)}{1+\exp\left(\mu_{0}+\sum \beta X_{i}\right)},\theta =\exp\left(\theta^{\prime }\right).$$

Let *n_p_* denote the subset of couples with observed pregnancies, *n_c_* the subset of couples that were right censored, *n* = *n_c_* + *n_p_* the total sample size and *T_i_* the last observed cycle for an individual couple *i* either corresponding to pregnancy or censoring. We can estimate the coefficients *β, θ* and the intercept *μ*_0_ for *X_ _= *0 via maximum likelihood optimization. The likelihood contribution of individual couples can be assumed to be independent, thus the total log‐likelihood *L* is the sum of the log‐likelihood for couples with observed pregnancies (*L_p_*) and the log‐likelihood for couples who were right‐censored (*L_c_*). After rearrangement of the notation from Weinberg and Gladen (1986), we get

$$L\left(X_{1}\ldots X_{n},T_{1}\ldots T_{n},\mu_{0},\beta ,\theta \right)=\sum_{i\;\in \;n_{p}}L_{pi}+\sum_{i\;\in \;n_{c}}L_{ci},$$

where

$$L_{pi}\left(X_{i},T_{i},\mu_{0},\beta ,\theta \right)=\begin{array}{c} \ln\left(\mu (X_{i})\right)-\ln\left(1-\mu \left(X_{i}\right)+\left(T_{i}-1\right)\theta \right)\\ +\;\sum_{j=0}^{T_{i}-1}\left[\ln\left(1-\mu (X_{i})+j\theta \right)-\ln\left(1+j\theta \right)\right] \end{array}$$

and

(9) $$L_{ci}\left(X_{i},T_{i},\mu_{0},\beta ,\theta \right)=\sum_{j=0}^{T_{i}-1}\left[\ln\left(1-\mu (X_{i})+j\theta \right)-\ln\left(1+j\theta \right)\right].$$

After estimation of the parameters, dynamic predictions of the cumulative probability of pregnancy over prediction window *w* given *s* failed cycles can be calculated using Equation (7) and plugging in $\hat{\mu }$(*X_i_*) and $\hat{\theta }$.

The beta‐geometric model uses all data from inclusion to the end of follow‐up in its fit and is thus expected to be less sensitive to a small sample at later *s* than landmarking. Since it is parametric, it could be sensitive to misspecification, in addition to being limited to discrete time‐to‐event and baseline covariates. The closed expression prediction formula is convenient to apply since a small number of parameters are required to be able to calculate a prediction.

The beta‐geometric model can be extended to allow for a fraction of couples that have zero chances to conceive, that is absolute sterile couples (Weinberg & Gladen, 1986). This fraction, denoted as the sterility parameter *π*, can be estimated in the beta‐geometric model (Weinberg & Gladen, 1986). This is referred to as the mixture model. We extend the log‐likelihood function from Equation (9) to

(10) $$\begin{array}{ccc} & & L_{mixture}\;\left(X_{1}\ldots X_{n},T_{1}\ldots T_{n},\mu_{0},\beta ,\theta ,\pi \right)\\ & & \;=\sum_{i\;\in \;n_{p}}\left[\ln\left(1-\pi \right)+L_{pi}\right]+\sum_{i\;\in \;n_{c}}\left[\ln(\pi +\left(1-\pi \right)\;\exp\left(L_{ci}\right))\right]. \end{array}$$

After estimation of the parameters, again using maximum likelihood, the calculation of a probability to conceive within *s + w* cycles given that the couple did not conceive within *s* cycles now becomes

(11) $$P\left(T\leq s+w\;|\;T>s,X_{i}\right)=\left(1-\pi_{s}\right)\left(1-\prod_{j=s}^{s+w-1}\frac{1-\mu (X_{i})+j\theta }{1+j\theta }\right),$$

with $\pi_{s}$ denoting the expected fraction of couples that are sterile after *s* failed cycles

(12) $$\pi_{s}=\frac{\pi }{\pi +\left(1-\pi \right)\frac{1}{n}\sum_{i=1}^{n}\left(1-\prod_{j=0}^{s}\frac{1-\mu (X_{i})+j\theta }{1+j\theta }\right)}.$$

We described landmarking with three approaches to build a prediction model: fitting separate Cox models (model **A**), using landmarks as strata (model **B**) and incorporating linear and quadratic terms of landmark numbers as covariates (model **C**). We also described the beta‐geometric model with (model **D**, referred to as a mixture model) and without (model **E**) the sterility parameter.

Table Supp‐I in the Supporting Information summarizes the expected advantages and disadvantages of the five models.

We continue with describing the framework for the simulation study where we compare the performance of models A to E.

## 3 SIMULATION STUDY

The parameters of the simulation scenarios were closely based on what was observed in two recent cohorts following unexplained subfertile couples for natural conception (van der Steeg et al., 2007; van Eekelen et al., 2017a, 2018).

We are interested in the ability of the models to predict P(T≤s+w|T⟩s), not only from baseline (*s* = 0), but also after *s* failed cycles with *s* ∈ {0,1,2,…26}. We choose a prediction window *w* = 13 cycles, so we predict one year pregnancy chances from 27 time points onwards.

We compare the accuracy of the predictions from models A to E where we prefer low bias and high precision summarized in terms of the lowest root mean squared error of average predictions (RMSE) and the lowest root mean squared error of individual predictions (RMSPE). In addition, we were interested in other commonly used measures to denote and compare clinical prediction model performance, namely those that describe (internal) prediction error and discrimination: the Brier score and the c‐statistic (Steyerberg, 2009).

We simulate data by 10 different data‐generating mechanisms to assess the robustness of models over multiple scenarios.

### 3.1 Data generation

We chose a discrete time to event because the beta‐geometric model is based on discrete cycles, not on calendar time.

To generate the time to event for each of *n *= 6000 couples, we followed these steps:

1. We simulated two covariates: female age in years (*X*_1_) drawn from a normal distribution *N*(34,5) and duration of subfertility (the number of years couples have been trying to conceive, *X*_2_) from an exponential distribution Exp(0.8)+1 such that the minimum for the latter was 1.
2. To gain covariate‐specific mean conception chances *μ*(*X_i_*), we combined the covariates with assumed linear effects on logit scale and used the intercept logit(*μ_0_*) of −0.60 such that
  (13) $$\mathrm{logit}\left(\mu (X_{i})\right)=\mathrm{logit}\left(\mu_{0}\right)-0.03*X_{1}*1\left[X_{1}\leq 33\right]-0.03*33*1\left[X_{1}>33\right]-0.15*\left(X_{1}-33\right)*1\left[X_{1}>33\right]-0.25*X_{2}$$
  and then backtransformed to means using the inverse‐logit.
3. To introduce heterogeneity, we assumed the individual probabilities followed a beta distribution with *μ*(*X_i_*) and *θ* from Equation (5) with fixed θ=0.15 to randomly draw an individual per cycle probability of pregnancy *p*(*X_i_*) for each couple. We consider other assumptions for the heterogeneity distribution later on.
4. We drew individual time to event values from a geometric distribution with success probability *p*(*X_i_*).
5. We assumed the censoring times were geometrically distributed with per cycle probability 0.1.
6. If the time of event was earlier or equal to the censoring time, the couple conceived during the study, otherwise they were followed until the time of censoring or the end of study, set at 52 cycles. Couples were followed for at least one cycle.

### 3.2 Model fitting

The two beta‐geometric models (models **D** and **E**) were fitted using Equations (10) and (9), respectively, by optimizing the log‐likelihood using the R command *optim*() and the Broyden–Fletcher–Goldfarb–Shanno (BFGS) algorithm based on Newton's gradient ascent. Predictions were calculated using Equations (11) and (12) for model D and Equation (7) for model E.

For the landmarking‐based models, we first derived all 27 landmark datasets by intervals of one cycle. We then followed the steps described in Chapter 2.1, selecting the subset still at risk at time *s* and censoring administratively at *s*+*w*.

We fitted separate Cox models using *cph*() on all landmarks (**A**) and derived predictions following Equation (1). After stacking all landmark datasets into one ‘super’ dataset, we fitted the ipl model (**B**) using *s* as strata and derived predictions as described before. We fitted the ipl* model (**C**) by removing the strata and adding linear and quadratic terms for *s* divided by 26 as covariates to the Cox model, then derived predictions following Equation (3).

In all models, we assumed a piecewise linear effect for age (differentiating between the effect of age below and above 33) and a linear effect for duration (*X_2_*).

In every landmark dataset, we calculated individual cumulative predicted probabilities of pregnancy $\hat{P}\left(T\leq s+13\;|\;T\right\rangle s,X_{i})$ using the fitted models A–E. We also calculated the true cumulative probability for every individual by using the generated individual probabilities *p*(*X_i_*) in every landmark dataset as

(14) $$P\left(T\leq s+13|T>s,X_{i}\right)=1-{\left(1-p\left(X_{i}\right)\right)}^{13}.$$

In addition to models A–E, we fitted a Kaplan–Meier curve (model **F**) for the sake of tracking the observed pregnancy rates and their variance between simulation replications as opposed to what is expected based on true probabilities.

### 3.3 Performance metrics

The following metrics were calculated at each landmark time point *s* where *n_s_* couples are still at risk.

We were interested in the expected error on the population level and the expected error on the individual level. For the first, to compare the average predictions based on a sample to the true population parameter, we averaged the individual cumulative predicted probabilities of pregnancy $\hat{P}$ into the average predicted probability $\hat{\bar{P}}$ For each simulated dataset, we averaged the generated individual cumulative probabilities *P* from Equation (14) to calculate the true average probability $\bar{P}$. We view the average of $\bar{P}$ over all simulation replications as the true (empirical) population parameter given all different, possible samples that could have been drawn. Bias of average predictions is defined as any deviation of $\hat{\bar{P}}$ in a replication from that simulation average of $\bar{P}$.

We then calculated the RMSE, that is the expected error in the average estimated probability denoted in percentage points compared to the true population parameter by

$$\mathrm{RMSE}=\sqrt{\frac{1}{nsim}\sum_{1}^{nsim}(\hat{\bar{P}}-{\left(\frac{1}{nsim}\sum_{1}^{nsim}\bar{P}\right)}^{2}}*100,$$

where *nsim* is the total number of simulation replications.

Next, we calculated the RMSPE, that is the expected error in individual predictions in percentage points by comparing it to the true individual probabilities per simulation replication following

$$\mathrm{RMSPE}\;=\sqrt{\frac{1}{nsim}\sum_{1}^{nsim}\left(\frac{1}{n_{s}}\sum_{i=1}^{n_{s}}{\left({\hat{P}}_{i}-P_{i}\right)}^{2}\right)}\;*100.$$

We derived Brier scores that represent the overall performance of the models, following Graf, Schmoor, Sauerbrei, and Schumacher (1999), and c statistics, that represent the discriminative ability of the models, following Harrell, Lee, and Mark (1996), both for right‐censored data. Finally, to show how sample size changes over time, we calculated the average of the sample size per landmark *n_s_* over all simulation replications.

In addition to results for models A–F, simulation metrics were also calculated using the true probabilities to attain the best obtainable (i.e. lowest) Brier score and best obtainable (i.e. maximum, highest) c statistic to aid interpretation of the differences of performance measures between models.

All metrics were evaluated on an external dataset, representing the out‐of‐sample performance of the models: For each development simulation set, a second validation set was generated using the same generating mechanism. The predicted probabilities in the validation set were calculated by applying the models that were fitted to the development set. True probabilities were based on the validation set only. We also evaluated the main scenario (scenario 1) on the training data, that is internal validation to see differences with out‐of‐sample performance.

### 3.4 Alternative simulation scenarios

We varied several aspects of the data generation algorithm that might influence the performance of the models.

First, the heterogeneity distribution could be different from beta: We used the compressed beta distribution and the logit‐normal distribution as alternatives. For the first, true probabilities *p*(*X*_i_) were drawn from a beta distribution as in step 5 of the main algorithm but multiplied with a compressor value of 0.6. For the second, individual log‐odds for pregnancy were drawn from a normal distribution $N(\mathrm{logit}(\mu (X_{i})),1)$ with adjusted logit(*μ*_0_) =−1.5. These were backtransformed to individual per cycle probabilities cycle $p(X_{i})$ using the inverse‐logit.

We also considered a scenario where there was no (unobserved) heterogeneity at all, so where the individual true probabilities per cycle were equal to the covariate‐specific means, that is $p\left(X_{i}\right)=\mu \left(X_{i}\right)$.

Second, we considered the possibility that a fraction of the couples in the cohort was sterile. To simulate this, we drew a random fraction of couples from a Bernoulli distribution with probability 0.3 (assuming 5% sterile couples in the population and the simulated data represent a subfertile cohort who have been trying to conceive for at least one year) and set their *p*(*X*_i_) to zero, meaning they are followed until the time of censoring or until the end of the study. The intercept *μ*_0_ was adjusted such that cumulative probabilities were similar to scenarios without the sterile fraction.

Third, it is assumed in the main scenario that for each couple, the true probabilities per cycle *p*(*X*_i_) are fixed over the entire follow‐up of the study. This may not hold, in particular because women age over follow‐up and their fecundability may decline. This is likely to have more impact in women that were older at the start of follow‐up (Sozou & Hartshorne, 2012).

We have implemented ageing in some of the simulation scenarios by assuming that *p*(*X*_i_) changed over time within the same couple. If the female age reached 33 years or above, their *p*(*X*_i_) decreased by the same 0.15 on logit scale that is assumed to hold for differences between couples in terms of baseline female age.

Finally, we considered the possibility where there is no censoring, for instance, in a retrospective study when data linkage with maternity databases is performed. For this scenario, we set the probability of right censoring per cycle to zero.

Table 1 summarizes all the 10 different scenarios we implemented.

**Table 1:** Description of scenarios. Parameters not included in this table did not differ between scenarios

Scenario number	Intercept logit(*μ_0_*)	Heterogeneity distribution	Ageing over follow‐up	Sterile fraction	Probability of censoring per cycle
1 (main)	−0.60	Beta	No	0.3	0.1
2 (no sterile fraction)	−1.05	Beta	No	0	0.1
3 (ageing over follow‐up)	−0.60	Beta	Yes	0.3	0.1
4 (no censoring)	−0.60	Beta	No	0.3	0
5 (no frailty)	−1.20	None	No	0.3	0.1
6 (no frailty and no sterile fraction)	−1.77	None	No	0	0.1
7 (logit normal frailty)	−1.5	Logit normal	No	0.3	0.1
8 (logit normal frailty and ageing)	−1.5	Logit normal	Yes	0.3	0.1
9 (compressed beta frailty)	−0.43	Compressed beta	No	0.3	0.1
10 (compressed beta frailty and ageing)	−0.43	Compressed beta	Yes	0.3	0.1

We consider scenario 1 as the main scenario because we view it as the most realistic given evidence from literature (Hunault et al., 2004; van der Steeg et al., 2007; van Eekelen et al., 2017a, 2018).

All simulations were replicated 1000 times. All datasets were generated using a known seed. We tracked how often models failed to converge. If so, results were calculated using replications where models did converge.

All analyses were performed using R version 3.3.2 (R Core Team [2017], R: A language and environment for statistical computing. R Foundation for Statistical Computing, Vienna, Austria. http://www.R-project.org/) using the *survival*, *dynpred*, *rms*, *xtable* and *ipred* packages.

Source code for all data generation, model fitting, simulation replication and processing of simulation results are provided online at the *Biometrical Journal* web page.

## 4 RESULTS

### 4.1 Bias in the average predictions

Results for the average predictions over all landmarks are visualized in Figure 1 for scenarios 1–10.

Figure 1Average predictions $\left(\hat{\bar{P}}(T\leq s+13|T>s,X_{i})\right)$ and true values ($\bar{P}\left(T\leq s+13|T\right\rangle s,X_{i}))$ for all landmarks *s* for scenarios 1–10

**Figure:**
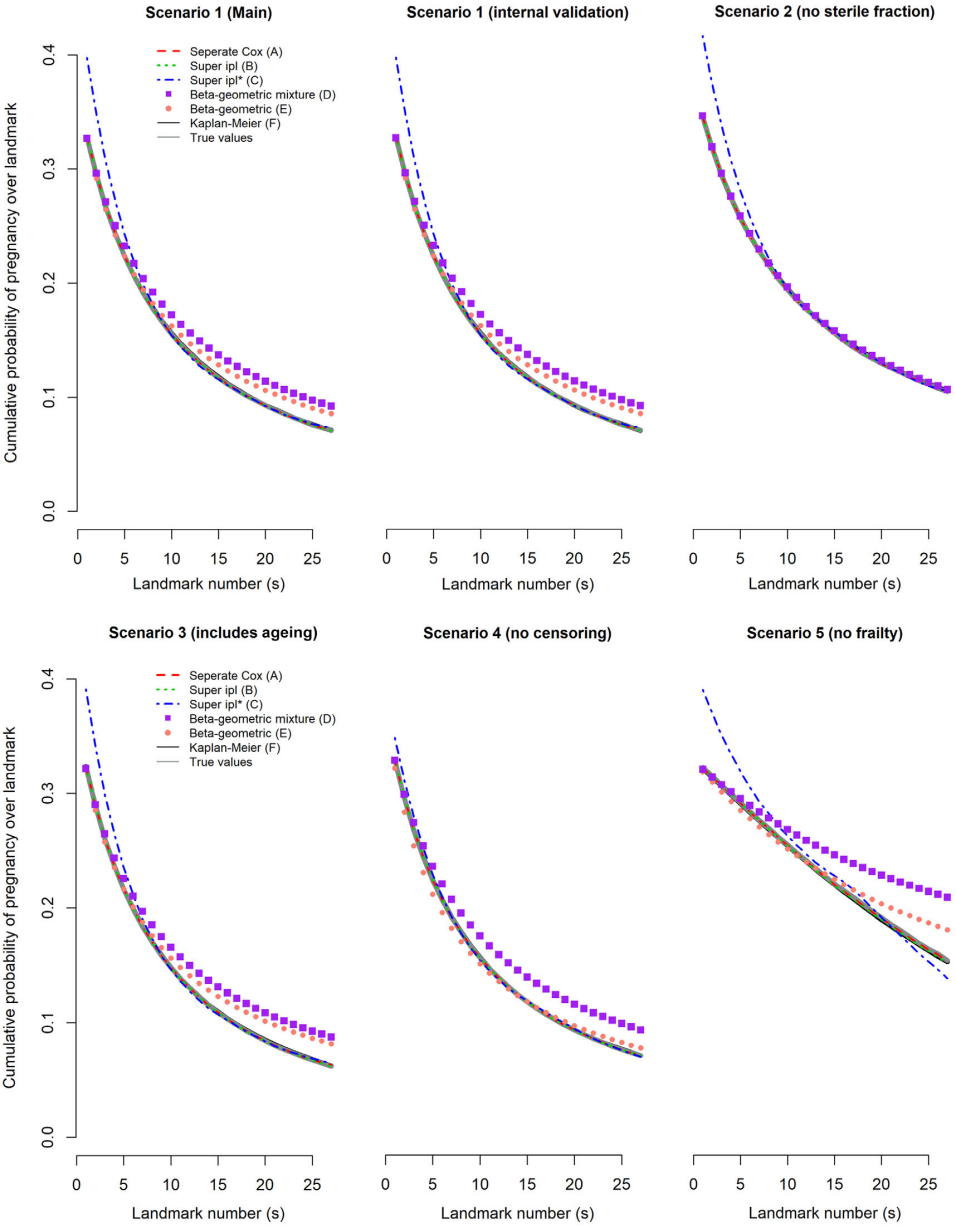

**Figure:**
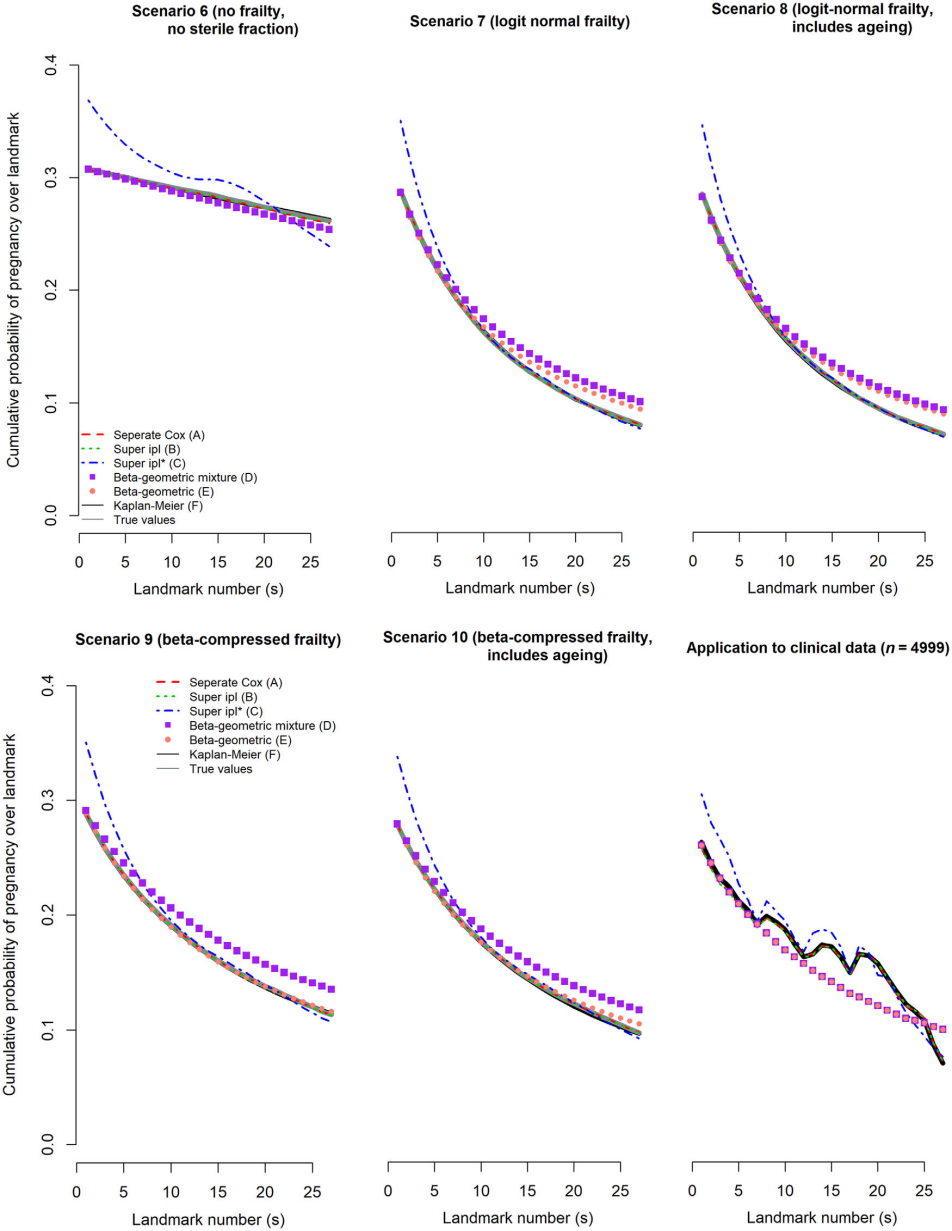

The ipl* model (C) overestimated the average cumulative probability of pregnancy for all scenarios up until landmark 10. This is likely due to an inaccurate approximation of the baseline hazard function using a linear/quadratic functional form in the model.

In the main scenario 1, all models except C (which overestimated by 6.9 percentage points in the baseline landmark) gave unbiased estimates of the average predictions until landmark 5 but both beta‐geometric models overestimated chances in landmarks thereafter (D by 2.1 percentage points and E by 1.5 percentage points in landmark 26). This is likely because the presence of a sterile fraction led to inaccuracy in the estimation of the beta distribution. Model D often had difficulty estimating the sterile fraction: Model D estimated an average fraction of sterile couples at baseline of 0.5% and in 24% of simulation replications this was less than 5%, compared to the true value of 30% used in the simulation. Results when the average prediction was evaluated on internal data were similar to the main scenario 1, which was expected given we were evaluating against the population parameter which is identical for internal and external datasets.

In the scenario without sterile couples (scenario 2), all models except model C were unbiased. Including ageing over follow‐up (scenario 3) yielded similar results as scenario 1, so all models were robust with regard to ageing.

In the scenario without censoring (scenario 4), the beta‐geometric mixture model (D) was better able to estimate the fraction of sterile couples (now 27% on average at baseline) but this did not improve accuracy of the average predictions of the model.

In the scenario where there was no heterogeneity (scenario 5), both beta‐geometric models performed poorly since they falsely assumed some degree of heterogeneity (estimates for *θ* were substantially lower compared to scenario 1 but non‐zero in all simulations). However, in the scenario where there was neither heterogeneity nor a fraction of sterile couples (scenario 6), we can see that both beta‐geometric models no longer found spurious heterogeneity (estimates for *θ* were almost zero). This falsely assumed heterogeneity in scenario 5 thus seems to result from the inability of the model to accurately estimate both the sterile fraction and the beta distribution.

In scenarios where we used the logit‐normal distribution for heterogeneity (scenarios 7 and 8, the latter with ageing) or the compressed beta distribution (scenarios 9 and 10, the latter with ageing), the results were similar to scenarios 1 and 3 although there was slightly more bias in the beta‐geometric mixture model (D) in scenarios 9 and 10. This indicates that the beta‐geometric models were robust to misspecification of the exact heterogeneity distribution.

### 4.2 RMSE in the average predictions

Table 2 denotes the RMSE for average predictions for *s* ∈ {0,13,26} for scenarios 1–10. Sample sizes per landmark were similar for all scenarios except for scenario 4 in which no censoring is present. In the main scenario 1, the RMSE increased for all models in later landmarks due to decreasing sample sizes. RMSEs at baseline were around 0.7–0.8 percentage points for all models except ipl* (C), which had a prediction error of approximately 7 percentage points due to its large bias. The beta‐geometric model without sterility parameter (E) performed the best in all landmarks except *s *= 0 in scenario 1 in terms of the lowest RMSE. This is because, albeit slightly biased upwards at the later landmarks, model E had the lowest empirical standard error, that is the highest precision. Results when the RMSE was evaluated on internal data were similar to the main scenario 1, which was expected given we were evaluating against the population parameter which is identical for internal and external datasets.

**Table 2:** RMSE (in percentage points) of average predictions for models A–F or the empirical variance of true probabilities between simulation replications in selected *s* for scenarios 1 to 10

Scenario	*s*	*n*	Separate Cox (A)	Super ipl (B)	Super ipl* (C)	Beta‐geometric mixture (D)	Beta‐geometric (E)	Kaplan–Meier (F)	Empirical variance
1 (main)	0	6000	0.826	0.831	7.03	0.727	0.759	0.825	0.481
	13	1023	1.42	1.41	1.69	2.20	1.21	1.42	0.68
	26	228	2.43	2.43	2.11	2.30	1.55	2.46	1.03
1 (internal validation)	0		0.823	0.829	7.04	0.727	0.755	0.825	0.489
	13		1.41	1.41	1.68	2.22	1.22	1.42	0.699
	26		2.42	2.43	2.10	2.35	1.57	2.46	0.953
2 (no sterile fraction)	0		0.815	0.82	7.18	0.738	0.715	0.827	0.451
	13		1.58	1.58	1.90	0.925	0.846	1.59	0.666
	26		3.02	2.97	2.61	0.802	0.749	2.96	1.08
3 (ageing over follow‐up)	0		0.797	0.796	6.84	0.725	0.76	0.788	0.477
	13		1.34	1.34	1.57	2.34	1.41	1.33	0.638
	26		2.15	2.17	1.91	2.65	1.99	2.19	0.879
4 (no censoring)	0	6000	0.603	0.619	2.09	0.548	0.903	0.614	0.468
	13	4031	0.523	0.523	0.526	2.20	0.308	0.516	0.331
	26	3531	0.417	0.418	0.415	2.28	0.697	0.412	0.247
5 (no frailty)	0		0.874	0.891	7.00	0.731	0.771	0.888	0.321
	13		1.86	1.85	2.34	2.92	1.28	1.87	0.726
	26		3.61	3.51	3.29	6.12	3.19	3.58	1.46
6 (no frailty and no sterile fraction)	0		0.843	0.844	6.23	0.68	0.68	0.859	0.166
	13		2.08	2.08	2.90	1.07	1.09	2.08	0.382
	26		4.61	4.55	4.66	1.77	1.78	4.62	0.904
7 (logit normal frailty)	0		0.793	0.795	6.35	0.739	0.732	0.788	0.43
	13		1.45	1.45	1.77	2.02	1.08	1.45	0.673
	26		2.36	2.32	2.02	2.43	1.59	2.34	1.02
8 (logit normal frailty and ageing)	0		0.768	0.769	6.20	0.737	0.746	0.758	0.426
	13		1.44	1.43	1.73	1.85	1.26	1.44	0.619
	26		2.28	2.22	1.91	2.39	1.92	2.26	0.879
9 (compressed beta frailty)	0		0.807	0.815	6.33	0.758	0.694	0.812	0.387
	13		1.58	1.57	1.92	2.62	0.935	1.60	0.644
	26		2.89	2.84	2.53	3.07	0.908	2.84	1.03
10 (compressed beta frailty and ageing)	0		0.788	0.79	6.12	0.713	0.684	0.793	0.383
	13		1.56	1.56	1.90	2.24	0.907	1.56	0.603
	26		2.79	2.78	2.41	2.75	1.17	2.79	0.968

In scenario 2 without sterile couples, both beta‐geometric models vastly outperformed the landmarking models because now both approaches were unbiased but the beta‐geometric models were more precise. In scenario 3 including ageing over follow‐up, the beta‐geometric models performed less well compared to scenario 1 but still outperformed the landmarking‐based Cox models in most landmarks in terms of the lowest RMSE. In scenario 4 without censoring, landmarking‐based Cox models A and B outperformed the beta‐geometric models D and E in terms of a lower RMSE because there was much more data available in later landmarks (*n_s_* = 3531 at *s* = 26 where it was approximately *n_s_* = 228 for scenarios 1–3). In scenarios 5–10, the beta‐geometric model without sterility parameter (E) performed best overall in terms of the lowest RMSE.

### 4.3 RMSPE in the individual predictions

Table 3 denotes the RMSPE for individual predictions for *s* ∈ {0,13,26} for scenarios 1–10. The RMSPE were very high (approximately 35% off for *s *= 0 in scenario 1) since the unobserved heterogeneity in individual chances of pregnancy far outweighed the information in the two simulated covariates, which also holds for the out‐of‐sample performance for scenario 1. The RMSPE decreased for all models in later landmarks due to a more homogeneous subset, and a larger sterile fraction, of couples that remain in the cohort. The only exception was scenario 6 with no heterogeneity and no sterile fraction of couples where the RMSPE was very low (approximately 1.5% at *s *= 0) and increased over landmarks.

**Table 3:** RMSPE (in percentage points) of individual predictions for models A–F or using true individual probabilities in selected *s* for scenarios 1–10

Scenario	*s*	*n*	Separate Cox (A)	Super ipl (B)	Super ipl* (C)	Beta‐geometric mixture (D)	Beta‐geometric (E)	Kaplan–Meier (F)	True probabilities
1 (main)	0	6000	35.2	35.2	36.0	35.2	35.2	37.1	0
	13	1023	21.4	21.3	21.3	21.5	21.3	21.6	0
	26	228	15.4	14.9	14.9	15.0	14.9	15.0	0
1 (internal validation)	0		35.2	35.2	35.9	35.2	35.2	37.1	0
	13		21.3	21.3	21.3	21.5	21.3	21.6	0
	26		15.0	14.7	14.7	14.9	14.7	14.8	0
2 (no sterile fraction)	0		31.9	31.9	32.7	31.9	31.9	34.6	0
	13		20.7	20.6	20.6	20.5	20.5	21.9	0
	26		16.1	15.2	15.2	14.9	14.9	16.0	0
3 (ageing over follow‐up)	0		34.9	34.9	35.6	34.9	34.9	36.8	0
	13		20.3	20.2	20.2	20.4	20.2	20.5	0
	26		13.9	13.3	13.3	13.5	13.4	13.4	0
4 (no censoring)	0	6000	35.2	35.3	35.3	35.2	35.2	37.0	0
	13	4031	21.2	21.2	21.2	21.5	21.2	21.6	0
	26	3531	14.6	14.6	14.6	15.0	14.6	14.7	0
5 (no frailty)	0		22.6	22.6	23.7	22.6	22.6	25.4	0
	13		23.3	23.3	23.4	23.6	23.4	24.0	0
	26		21.7	21.6	21.5	22.9	22.0	21.1	0
6 (no frailty and no sterile fraction)	0		1.5	1.6	6.8	1.3	1.3	12.7	0
	13		3.6	2.5	3.4	1.5	1.5	12.8	0
	26		8.4	5.0	5.1	1.9	1.9	13.2	0
7 (logit normal frailty)	0		31.5	31.5	32.2	31.5	31.5	33.2	0
	13		21.2	21.1	21.2	21.3	21.1	21.6	0
	26		15.9	15.3	15.3	15.5	15.3	15.4	0
8 (logit normal frailty and ageing)	0		31.1	31.1	31.8	31.1	31.1	32.9	0
	13		20.0	20.0	20.0	20.0	20.0	20.5	0
	26		14.4	13.8	13.8	13.9	13.8	14.0	0
9 (compressed beta frailty)	0		28.1	28.1	28.8	28.1	28.1	29.5	0
	13		20.7	20.6	20.7	20.9	20.7	20.9	0
	26		17.2	16.7	16.6	17.0	16.5	16.6	0
10 (compressed beta frailty and ageing)	0		27.7	27.7	28.4	27.7	27.7	29.2	0
	13		19.4	19.3	19.4	19.5	19.3	19.7	0
	26		15.4	14.7	14.6	14.9	14.5	14.7	0

Aside from the ipl* model (C) that generally performed the worst in the first landmark *s *= 0 in most scenarios and the beta‐geometric models that performed the best in scenario 6 without heterogeneity, there were no relevant differences between models suggesting that all models were similar in their (in)ability to estimate individual predictions in the presence of substantial unobserved heterogeneity.

### 4.4 Brier score

All models had similar Brier scores in scenarios 1–10 (Supporting Information, Table Supp‐III) with no model clearly being preferred over another. The Brier score for the Kaplan–Meier (F) represents the prediction error for a model without covariates and was close to the Brier scores of models A–E for all scenarios since the unobserved heterogeneity in individual chances of pregnancy far outweighed the information in the two simulated covariates.

### 4.5 c statistic

The c statistics (Supporting Information, Table Supp‐IV) were also similar for all models in scenarios 1–10 except for the separate Cox approach (A). This approach yielded the poorest statistic in nearly all scenarios and landmarks, except in scenario 1 where the c statistic was evaluated on internal data. In that scenario, the separate Cox landmark approach (model A) yielded the highest c statistic beyond landmark s = 0, with a value of 0.66 in landmark s = 26. This indicates that model A might discriminate well in the development data landmarks but does not transport well to external datasets at later landmark time points.

### 4.6 Convergence

The beta‐geometric mixture model (D) failed to converge in 17.7% of simulation replications of the main scenario 1 and at least one of the separate Cox landmark models (model A) in 2.3%. The other models always converged. Model D failed due to insufficient information after censoring and an insufficient length of follow‐up to estimate the sterile fraction and model A failed due to an insufficient number of events in later landmarks. Table Supp‐II in the Supporting Information shows the proportion of convergence failure for models D and A in the remaining simulation scenarios.

## 5 APPLICATION

We applied the five models to clinical data obtained from 2002 to 2004 in a Dutch national prospective cohort study conducted in 38 fertility centres (van der Steeg et al., 2007; van Eekelen et al., 2017a). Couples for which no barrier to pregnancy could be found during the fertility workup (unexplained subfertility) were followed for pregnancy from the completion of the fertility workup onwards. Data on *n* = 4999 couples were available, which decreased to *n* = 151 in the final landmark. The median number of cycles of follow‐up was 7. After fitting the models, we evaluated the average predictions, Brier scores and c statistics in all landmarks in an internal validation with all methods and approaches similar to the simulation study.

To align with the simulation study, we only used the covariates female age and duration of subfertility in the models as these were shown to be the most important predictors of pregnancy in previous work, as was the piecewise linear effect of female age above and below approximately 33 years (Hunault et al., 2004). All models converged. The model parameters are shown in Table 4 for the landmarking‐based models and Table 5 for the beta‐geometric models.

**Table 4:** Estimated parameters for the landmarking‐based models in the data application

Parameter	Separate Cox (A)	Super ipl (B)	Super ipl* (C)
Coefficient for age < 33 years	Varied from −0.12 to −0.01	−0.04	−0.04
Coefficient for age > 33 years	Varied from −0.15 to 0.02	−0.08	−0.08
Coefficient for duration	Varied from −0.44 to −0.17	−0.23	−0.23
Coefficient for linear term for baseline hazard	–	–	−5.67
Coefficient for squared term for baseline hazard	–	–	1.50

**Table 5:** Estimated parameters for the beta‐geometric models in the data application

Parameter	Beta‐geometric mixture (D)	Beta‐geometric (E)
Coefficient for age < 33 years	−0.03	−0.03
Coefficient for age > 33 years	−0.08	−0.08
Coefficient for duration	−0.29	−0.29
Intercept (μ_0_)	0.17	0.17
Heterogeneity (θ)	0.11	0.11
Sterile fraction (π)	0.002	–

The pooled estimates for coefficients from the ‘stacked’ landmarking‐based models (B and C) are easier to interpret than the separate Cox models as for the latter, the coefficients change over time. We observed that some coefficients in models B and C pointed in counterintuitive directions such as a positive effect of increasing female age over 33 years on pregnancy. The estimated sterile fraction in the beta‐geometric mixture model (model D) was close to zero.

The average model predictions per landmark in the application to clinical data are shown in the last, lower right‐most panel of Figure 1, decreasing from 0.27 in *s *= 0 to 0.07 in *s = *26. Notably, the tendency of the super ipl* landmark model (model C) to overestimate in the first six landmarks compared to the Kaplan–Meier estimates was also visible in this application to real data. The beta‐geometric models underestimated compared to the Kaplan–Meier estimates from landmark *s *= 7 onwards, but due to the rapidly decreasing sample size over time Kaplan–Meier estimates were imprecise. Estimates from Brier scores were similar for all models, decreasing from 0.21 in *s *= 0 to 0.07 in *s = *26. c statistics were also similar for all models and comparable to fixed, that is non‐dynamic models in the field, but showed more variability over landmarks than Brier scores, with a median of 0.60, increasing from 0.60 in *s *= 0 to the highest value of 0.71 in *s *= 24, back to a value of 0.59 in *s = *26.

## 6 DISCUSSION

In this paper, we have compared two methods for dynamic prediction of time to pregnancy and applied these to clinical data. We observed in most of our simulation scenarios that although the beta‐geometric models were slightly biased at later landmarks, the beta‐geometric model without the sterility parameter had the lowest RMSE of average predictions out of all models due to higher precision. The landmarking‐based Cox models performed better than the beta‐geometric models in terms of RMSE when a large amount of information, in terms of sample size in both the baseline landmark and in later landmarks, was available. All models were comparable in RMSPE and Brier scores due to the difficulty of estimating chances on the individual level in the presence of a strong degree of unobserved heterogeneity between couples. The separate Cox approach (A) yielded a high internal c statistic at later landmarks in internal validation but did not transport well to external data due to following noise in highly varying, small landmark risk sets later on.

Our results were based on a simulation study using several data generating mechanisms, but there remain limitations to interpretations and generalizations when applying these methods to clinical data of which the data‐generating mechanisms are unknown.

The super ipl* landmark model was not able to accurately model the baseline hazard in the early landmarks, not even when adding cubic terms of landmark numbers or when using natural or restricted cubic splines of landmark numbers as a sensitivity analysis, so we advise against using this method. The tendency of this model to overestimate chances in early landmarks was also noted in the internal validation of our application to clinical data. The main challenge for the other two landmarking approaches is a low (effective) sample size, not only expressed as the risk set in the first landmark but in particular if one wishes to cover later landmarks in the presence of censoring. When estimating separate Cox models on all landmarks, the fluctuations in covariate effects over time are sensitive to noise in small risk sets. Another disadvantage of this method is that varying covariate effects can be difficult to report and communicate to clinicians and their patients.

We recommend landmarking in combination with fitting a super ipl Cox model that incorporates landmarks as strata when the effective sample size remains above 500 over follow‐up since this model was both accurate and concise. At sample sizes around 500 or lower, the RMSE for the model increased considerably.

Both beta‐geometric models were sensitive to the presence of a sterile fraction which led to overestimated probabilities at later landmarks. Estimation of the sterile fraction is computationally challenging and convergence requires sufficient information, both in terms of a large sample size and a long follow‐up (Klebanov & Yakovlev, 2007; Shi & Yin, 2017). In our application to clinical data, the sterility parameter was close to zero, which might be due to couples in the data being followed for only a median of seven cycles. If the effective sample size drops below 500 in landmarks that researchers wish to cover, as was the case in the data used by van Eekelen et al. (2017a) to develop their clinical prediction model, the beta‐geometric model without the sterility parameter is a more precise alternative than landmarking. The beta‐geometric model was also robust across scenarios where the heterogeneity distribution was not strictly beta or where we introduced decreasing probabilities of conception per cycle due to ageing over follow‐up.

The beta‐geometric model can also be useful in other fields where there is a discrete time to event. Other examples could be the number of days to discharge from an intensive care unit, the number of payments to defaulting for mortgages or time to unsubscription for companies offering services in monthly subscriptions such as cell phone plans. However, there may be time‐varying information that researchers wish to incorporate in predictions such as divorce which increases the probability to default on a mortgage, something the beta‐geometric model is not capable of. Thus, another reason to prefer landmarking methods in some situations is that they are more flexible in incorporating time‐varying covariates.

## CONFLICT OF INTEREST

The authors have declared no conflict of interest.

## Supporting information

Supporting Information

Supporting Information
