# Supplementary material for: A comparison of the beta‐geometric model with landmarking for dynamic prediction of time to pregnancy
Source: Biom J. 2019 Nov 18;62(1):175–90. doi: 10.1002/bimj.201900155 (PMC6973003; doi:10.1002/bimj.201900155)
Supplement: Supplementary file 1 — Supporting Information [file BIMJ-62-175-s002.docx]

**SUPPLEMENTARY MATERIAL**

**Table Supp-I**. Expected advantages and disadvantages of the five models

| **Method** | **Model** | **Pros** |  | **Cons** |  |
| --- | --- | --- | --- | --- | --- |
|  |  |  |  |  |  |
| **Landmarking** | **A. Separate Cox models for all landmarks** | Semi-parametric  Flexible  Allows regression coefficients to change over landmarks | | All estimates are sensitive to small sample sizes in later landmarks  Lots of information to report  Proportional hazards assumption | |
|  | **B. Super Cox model with landmark datasets as strata (ipl)** | Semi-parametric  Flexible  Estimates one set of regression coefficients | | Baseline hazard is sensitive to sample size in later landmarks  Lots of baseline hazards to estimate and report  Proportional hazards assumption | |
|  | **C. Super Cox model with landmark datasets as covariates (ipl*)** | Less baseline hazards need to be estimated  Estimates one set of regression coefficients  Allows predictions from any landmark until the maximum follow up | | Partly parametric, the function for baseline hazards could be misspecified  Proportional hazards assumption | |
| **Beta-geometric** | **D. Beta-geometric mixture model with the parameter π for the sterile fraction** | Fully parametric  Uses all data at once  Estimates one set of coefficients  Yields a concise, closed expression for predictions  Estimates π that may be of interest | | Sensitive to misspecification  Convergence issues  Proportional odds assumption  Limited to baseline covariates and discrete time-to-event | |
|  | **E. Beta-geometric model without sterile fraction parameter** | Fully parametric  Uses all data at once  Estimates one set of coefficients  Yields a concise, closed expression for predictions | | Sensitive to misspecification  Proportional odds assumption  Limited to baseline covariates and discrete time-to-event | |

**Table Supp-II.** Percentage of simulation replications where a model failed to converge

| Scenario | Percentage of failed convergences (model A, only occurred at s = 22 to 26) | Percentage of failed convergences (model D) |
| --- | --- | --- |
| 1 | 2.3% | 17.7% |
| 2 | 0.4% | 14.3% |
| 3 | 3% | 6.4% |
| 4 | 0 | 0 |
| 5 | 0.2% | 0.2% |
| 6 | 0 | 0.2% |
| 7 | 1.1% | 8.4% |
| 8 | 1.1% | 2.8% |
| 9 | 0 | 7.6% |
| 10 | 0.3% | 10.7% |

**Tables Supp-III. Brier scores for predictions from models A through F or the best obtainable using true probabilities in selected *s* for scenarios 1 to 10.**

| Scenario | ***s*** | **n** | **Separate Cox**  **(A)** | **Super ipl (B)** | **Super ipl* (C)** | **Beta-geometric mixture**  **(D)** | **Beta-geometric (E)** | **Kaplan-Meier (F)** | **Best obtainable** |
| --- | --- | --- | --- | --- | --- | --- | --- | --- | --- |
| 1 (main) | 0 | 6000 | 0.222 | 0.222 | 0.224 | 0.222 | 0.222 | 0.236 | 0.088 |
|  | 13 | 1023 | 0.117 | 0.117 | 0.117 | 0.117 | 0.117 | 0.119 | 0.066 |
|  | 26 | 228 | 0.074 | 0.073 | 0.072 | 0.072 | 0.072 | 0.073 | 0.048 |
| 1 (internal validation) | 0 |  | 0.221 | 0.221 | 0.223 | 0.221 | 0.221 | 0.236 | 0.088 |
|  | 13 |  | 0.116 | 0.116 | 0.116 | 0.116 | 0.116 | 0.118 | 0.066 |
|  | 26 |  | 0.070 | 0.071 | 0.071 | 0.073 | 0.072 | 0.072 | 0.048 |
| 2 (no sterile fraction) | 0 |  | 0.222 | 0.222 | 0.224 | 0.222 | 0.222 | 0.242 | 0.113 |
|  | 13 |  | 0.142 | 0.141 | 0.141 | 0.141 | 0.141 | 0.148 | 0.094 |
|  | 26 |  | 0.103 | 0.101 | 0.100 | 0.100 | 0.100 | 0.104 | 0.075 |
| 3 (aging over follow up) | 0 |  | 0.220 | 0.220 | 0.222 | 0.220 | 0.220 | 0.235 | 0.088 |
|  | 13 |  | 0.111 | 0.110 | 0.111 | 0.111 | 0.110 | 0.112 | 0.064 |
|  | 26 |  | 0.065 | 0.064 | 0.064 | 0.064 | 0.064 | 0.064 | 0.044 |
| 4 (no censoring) | 0 | 6000 | 0.207 | 0.208 | 0.208 | 0.207 | 0.207 | 0.221 | 0.083 |
|  | 13 | 4031 | 0.107 | 0.107 | 0.107 | 0.108 | 0.107 | 0.109 | 0.062 |
|  | 26 | 3531 | 0.066 | 0.066 | 0.066 | 0.067 | 0.066 | 0.066 | 0.044 |
| 5 (no frailty) | 0 |  | 0.219 | 0.219 | 0.221 | 0.219 | 0.219 | 0.234 | 0.163 |
|  | 13 |  | 0.187 | 0.187 | 0.187 | 0.188 | 0.187 | 0.191 | 0.126 |
|  | 26 |  | 0.146 | 0.146 | 0.145 | 0.149 | 0.146 | 0.143 | 0.092 |
| 6 (no frailty and no sterile fraction) | 0 |  | 0.211 | 0.211 | 0.212 | 0.211 | 0.211 | 0.228 | 0.210 |
|  | 13 |  | 0.203 | 0.202 | 0.202 | 0.202 | 0.202 | 0.220 | 0.202 |
|  | 26 |  | 0.200 | 0.195 | 0.196 | 0.193 | 0.193 | 0.212 | 0.192 |
| 7 (logit normal frailty) | 0 |  | 0.209 | 0.209 | 0.210 | 0.209 | 0.209 | 0.221 | 0.100 |
|  | 13 |  | 0.125 | 0.124 | 0.125 | 0.125 | 0.124 | 0.127 | 0.074 |
|  | 26 |  | 0.083 | 0.082 | 0.081 | 0.082 | 0.081 | 0.082 | 0.054 |
| 8 (logit normal frailty and aging) | 0 |  | 0.206 | 0.206 | 0.208 | 0.206 | 0.206 | 0.219 | 0.100 |
|  | 13 |  | 0.117 | 0.117 | 0.117 | 0.117 | 0.117 | 0.119 | 0.072 |
|  | 26 |  | 0.074 | 0.073 | 0.073 | 0.073 | 0.072 | 0.073 | 0.051 |
| 9 (compressed beta frailty) | 0 |  | 0.212 | 0.212 | 0.213 | 0.211 | 0.211 | 0.221 | 0.125 |
|  | 13 |  | 0.150 | 0.150 | 0.150 | 0.150 | 0.150 | 0.151 | 0.102 |
|  | 26 |  | 0.114 | 0.112 | 0.112 | 0.112 | 0.111 | 0.111 | 0.080 |
| 10 (compressed beta frailty and aging) | 0 |  | 0.208 | 0.209 | 0.210 | 0.208 | 0.208 | 0.218 | 0.124 |
|  | 13 |  | 0.139 | 0.139 | 0.139 | 0.139 | 0.138 | 0.140 | 0.097 |
|  | 26 |  | 0.100 | 0.098 | 0.098 | 0.097 | 0.097 | 0.098 | 0.073 |

**Tables Supp-IV. C statistic for models A through F and best obtainable using true probabilities in selected *s* for scenarios 1 to 10.**

| Scenario | ***s*** | **n** | **Separate Cox**  **(A)** | **Super ipl (B)** | **Super ipl* (C)** | **Beta-geometric mixture**  **(D)** | **Beta-geometric (E)** | **Kaplan-Meier (F)** | **Best obtainable** |
| --- | --- | --- | --- | --- | --- | --- | --- | --- | --- |
| 1 (main) | 0 | 6000 | 0.633 | 0.632 | 0.632 | 0.633 | 0.633 | 0.500 | 0.892 |
|  | 13 | 1023 | 0.592 | 0.600 | 0.600 | 0.601 | 0.601 | 0.500 | 0.915 |
|  | 26 | 228 | 0.542 | 0.583 | 0.583 | 0.582 | 0.583 | 0.500 | 0.928 |
| 1 (internal validation) | 0 |  | 0.634 | 0.633 | 0.633 | 0.634 | 0.634 | 0.500 | 0.892 |
|  | 13 |  | 0.611 | 0.602 | 0.602 | 0.602 | 0.601 | 0.500 | 0.916 |
|  | 26 |  | 0.656 | 0.578 | 0.578 | 0.576 | 0.577 | 0.500 | 0.925 |
| 2 (no sterile fraction) | 0 |  | 0.642 | 0.642 | 0.642 | 0.642 | 0.642 | 0.500 | 0.866 |
|  | 13 |  | 0.642 | 0.646 | 0.646 | 0.646 | 0.646 | 0.500 | 0.869 |
|  | 26 |  | 0.617 | 0.652 | 0.652 | 0.651 | 0.652 | 0.500 | 0.871 |
| 3 (aging over follow up) | 0 |  | 0.634 | 0.634 | 0.634 | 0.634 | 0.634 | 0.500 | 0.892 |
|  | 13 |  | 0.601 | 0.608 | 0.608 | 0.609 | 0.608 | 0.500 | 0.915 |
|  | 26 |  | 0.555 | 0.593 | 0.593 | 0.592 | 0.594 | 0.500 | 0.927 |
| 4 (no censoring) | 0 | 6000 | 0.628 | 0.628 | 0.628 | 0.628 | 0.628 | 0.500 | 0.896 |
|  | 13 | 4031 | 0.595 | 0.596 | 0.596 | 0.597 | 0.597 | 0.500 | 0.917 |
|  | 26 | 3531 | 0.574 | 0.577 | 0.577 | 0.577 | 0.577 | 0.500 | 0.928 |
| 5 (no frailty) | 0 |  | 0.635 | 0.634 | 0.634 | 0.635 | 0.635 | 0.500 | 0.765 |
|  | 13 |  | 0.596 | 0.600 | 0.600 | 0.601 | 0.600 | 0.500 | 0.823 |
|  | 26 |  | 0.527 | 0.547 | 0.547 | 0.547 | 0.547 | 0.500 | 0.872 |
| 6 (no frailty and no sterile fraction) | 0 |  | 0.642 | 0.642 | 0.642 | 0.642 | 0.642 | 0.500 | 0.643 |
|  | 13 |  | 0.648 | 0.650 | 0.650 | 0.650 | 0.650 | 0.500 | 0.650 |
|  | 26 |  | 0.646 | 0.661 | 0.661 | 0.661 | 0.661 | 0.500 | 0.661 |
| 7 (logit normal frailty) | 0 |  | 0.630 | 0.630 | 0.630 | 0.630 | 0.630 | 0.500 | 0.876 |
|  | 13 |  | 0.600 | 0.606 | 0.606 | 0.607 | 0.607 | 0.500 | 0.902 |
|  | 26 |  | 0.555 | 0.593 | 0.593 | 0.595 | 0.594 | 0.500 | 0.917 |
| 8 (logit normal frailty and aging) | 0 |  | 0.632 | 0.632 | 0.632 | 0.632 | 0.632 | 0.500 | 0.876 |
|  | 13 |  | 0.611 | 0.617 | 0.617 | 0.617 | 0.617 | 0.500 | 0.902 |
|  | 26 |  | 0.573 | 0.612 | 0.612 | 0.613 | 0.613 | 0.500 | 0.917 |
| 9 (compressed beta frailty) | 0 |  | 0.620 | 0.619 | 0.619 | 0.620 | 0.620 | 0.500 | 0.847 |
|  | 13 |  | 0.566 | 0.574 | 0.574 | 0.575 | 0.575 | 0.500 | 0.855 |
|  | 26 |  | 0.517 | 0.542 | 0.542 | 0.544 | 0.542 | 0.500 | 0.871 |
| 10 (compressed beta frailty and aging) | 0 |  | 0.622 | 0.622 | 0.622 | 0.622 | 0.622 | 0.500 | 0.847 |
|  | 13 |  | 0.582 | 0.588 | 0.588 | 0.589 | 0.588 | 0.500 | 0.857 |
|  | 26 |  | 0.532 | 0.565 | 0.565 | 0.566 | 0.566 | 0.500 | 0.872 |
