# Supplementary material for: A comparison of the beta‐geometric model with landmarking for dynamic prediction of time to pregnancy
Source: Biom J. 2019 Nov 18;62(1):175–90. doi: 10.1002/bimj.201900155 (PMC6973003; doi:10.1002/bimj.201900155)
Supplement: Supplementary file 2 — Supporting Information [file BIMJ-62-175-s001.zip › Code/tabAUC_1.html]

|  | 1 | 2 | 3 | 4 | 5 | 6 | 7 |
| --- | --- | --- | --- | --- | --- | --- | --- |
| 1 | 0.633 | 0.633 | 0.633 | 0.633 | 0.633 | 0.500 | 0.892 |
| 2 | 0.590 | 0.598 | 0.598 | 0.601 | 0.599 | 0.500 | 0.915 |
| 3 | 0.548 | 0.578 | 0.578 | 0.570 | 0.578 | 0.500 | 0.925 |
