# Supplementary material for: A comparison of the beta‐geometric model with landmarking for dynamic prediction of time to pregnancy
Source: Biom J. 2019 Nov 18;62(1):175–90. doi: 10.1002/bimj.201900155 (PMC6973003; doi:10.1002/bimj.201900155)
Supplement: Supplementary file 2 — Supporting Information [file BIMJ-62-175-s001.zip › Code/tabBr_2.html]

|  | 1 | 2 | 3 | 4 | 5 | 6 | 7 |
| --- | --- | --- | --- | --- | --- | --- | --- |
| 1 | 0.222 | 0.222 | 0.224 | 0.222 | 0.222 | 0.242 | 0.113 |
| 2 | 0.143 | 0.142 | 0.142 | 0.143 | 0.142 | 0.149 | 0.095 |
| 3 | 0.099 | 0.097 | 0.097 | 0.096 | 0.096 | 0.100 | 0.071 |
