# Supplementary material for: A comparison of the beta‐geometric model with landmarking for dynamic prediction of time to pregnancy
Source: Biom J. 2019 Nov 18;62(1):175–90. doi: 10.1002/bimj.201900155 (PMC6973003; doi:10.1002/bimj.201900155)
Supplement: Supplementary file 2 — Supporting Information [file BIMJ-62-175-s001.zip › Code/tabBr_6.html]

|  | 1 | 2 | 3 | 4 | 5 | 6 | 7 |
| --- | --- | --- | --- | --- | --- | --- | --- |
| 1 | 0.211 | 0.211 | 0.213 | 0.211 | 0.211 | 0.229 | 0.211 |
| 2 | 0.204 | 0.204 | 0.203 | 0.203 | 0.203 | 0.220 | 0.203 |
| 3 | 0.198 | 0.193 | 0.193 | 0.190 | 0.190 | 0.208 | 0.189 |
