# Supplementary material for: A comparison of the beta‐geometric model with landmarking for dynamic prediction of time to pregnancy
Source: Biom J. 2019 Nov 18;62(1):175–90. doi: 10.1002/bimj.201900155 (PMC6973003; doi:10.1002/bimj.201900155)
Supplement: Supplementary file 2 — Supporting Information [file BIMJ-62-175-s001.zip › Code/tabBr_8.html]

|  | 1 | 2 | 3 | 4 | 5 | 6 | 7 |
| --- | --- | --- | --- | --- | --- | --- | --- |
| 1 | 0.205 | 0.206 | 0.207 | 0.205 | 0.206 | 0.218 | 0.100 |
| 2 | 0.118 | 0.118 | 0.118 | 0.118 | 0.117 | 0.121 | 0.073 |
| 3 | 0.076 | 0.075 | 0.075 | 0.074 | 0.074 | 0.075 | 0.051 |
