# Supplementary material for: A comparison of the beta‐geometric model with landmarking for dynamic prediction of time to pregnancy
Source: Biom J. 2019 Nov 18;62(1):175–90. doi: 10.1002/bimj.201900155 (PMC6973003; doi:10.1002/bimj.201900155)
Supplement: Supplementary file 2 — Supporting Information [file BIMJ-62-175-s001.zip › Code/tabP_1.html]

|  | 1 | 2 | 3 | 4 | 5 | 6 | 7 | 8 |
| --- | --- | --- | --- | --- | --- | --- | --- | --- |
| 1 | 6000.000 | 0.328 | 0.328 | 0.397 | 0.326 | 0.326 | 0.328 | 0.328 |
| 2 | 1021.000 | 0.123 | 0.123 | 0.121 | 0.142 | 0.134 | 0.123 | 0.124 |
| 3 | 225.000 | 0.069 | 0.069 | 0.072 | 0.092 | 0.086 | 0.069 | 0.071 |
