# Supplementary material for: A comparison of the beta‐geometric model with landmarking for dynamic prediction of time to pregnancy
Source: Biom J. 2019 Nov 18;62(1):175–90. doi: 10.1002/bimj.201900155 (PMC6973003; doi:10.1002/bimj.201900155)
Supplement: Supplementary file 2 — Supporting Information [file BIMJ-62-175-s001.zip › Code/tabP_2.html]

|  | 1 | 2 | 3 | 4 | 5 | 6 | 7 | 8 |
| --- | --- | --- | --- | --- | --- | --- | --- | --- |
| 1 | 6000.000 | 0.345 | 0.345 | 0.417 | 0.346 | 0.345 | 0.345 | 0.345 |
| 2 | 992.000 | 0.163 | 0.163 | 0.163 | 0.164 | 0.163 | 0.163 | 0.164 |
| 3 | 211.000 | 0.099 | 0.099 | 0.100 | 0.107 | 0.106 | 0.099 | 0.107 |
