# Supplementary material for: A comparison of the beta‐geometric model with landmarking for dynamic prediction of time to pregnancy
Source: Biom J. 2019 Nov 18;62(1):175–90. doi: 10.1002/bimj.201900155 (PMC6973003; doi:10.1002/bimj.201900155)
Supplement: Supplementary file 2 — Supporting Information [file BIMJ-62-175-s001.zip › Code/tabP_3.html]

|  | 1 | 2 | 3 | 4 | 5 | 6 | 7 | 8 |
| --- | --- | --- | --- | --- | --- | --- | --- | --- |
| 1 | 6000.000 | 0.323 | 0.324 | 0.391 | 0.322 | 0.321 | 0.323 | 0.323 |
| 2 | 1026.000 | 0.116 | 0.116 | 0.112 | 0.137 | 0.129 | 0.115 | 0.115 |
| 3 | 228.000 | 0.065 | 0.063 | 0.066 | 0.087 | 0.082 | 0.063 | 0.061 |
