# Supplementary material for: A comparison of the beta‐geometric model with landmarking for dynamic prediction of time to pregnancy
Source: Biom J. 2019 Nov 18;62(1):175–90. doi: 10.1002/bimj.201900155 (PMC6973003; doi:10.1002/bimj.201900155)
Supplement: Supplementary file 2 — Supporting Information [file BIMJ-62-175-s001.zip › Code/tabP_4.html]

|  | 1 | 2 | 3 | 4 | 5 | 6 | 7 | 8 |
| --- | --- | --- | --- | --- | --- | --- | --- | --- |
| 1 | 6000.000 | 0.328 | 0.330 | 0.347 | 0.328 | 0.321 | 0.328 | 0.327 |
| 2 | 4033.000 | 0.124 | 0.124 | 0.124 | 0.145 | 0.123 | 0.124 | 0.123 |
| 3 | 3536.000 | 0.070 | 0.070 | 0.069 | 0.093 | 0.077 | 0.070 | 0.071 |
