# Supplementary material for: A comparison of the beta‐geometric model with landmarking for dynamic prediction of time to pregnancy
Source: Biom J. 2019 Nov 18;62(1):175–90. doi: 10.1002/bimj.201900155 (PMC6973003; doi:10.1002/bimj.201900155)
Supplement: Supplementary file 2 — Supporting Information [file BIMJ-62-175-s001.zip › Code/tabP_5.html]

|  | 1 | 2 | 3 | 4 | 5 | 6 | 7 | 8 |
| --- | --- | --- | --- | --- | --- | --- | --- | --- |
| 1 | 6000.000 | 0.325 | 0.326 | 0.393 | 0.324 | 0.321 | 0.325 | 0.323 |
| 2 | 1031.000 | 0.229 | 0.228 | 0.235 | 0.253 | 0.232 | 0.229 | 0.228 |
| 3 | 202.000 | 0.152 | 0.150 | 0.138 | 0.213 | 0.184 | 0.151 | 0.153 |
