# Supplementary material for: A comparison of the beta‐geometric model with landmarking for dynamic prediction of time to pregnancy
Source: Biom J. 2019 Nov 18;62(1):175–90. doi: 10.1002/bimj.201900155 (PMC6973003; doi:10.1002/bimj.201900155)
Supplement: Supplementary file 2 — Supporting Information [file BIMJ-62-175-s001.zip › Code/tabP_6.html]

|  | 1 | 2 | 3 | 4 | 5 | 6 | 7 | 8 |
| --- | --- | --- | --- | --- | --- | --- | --- | --- |
| 1 | 6000.000 | 0.307 | 0.307 | 0.369 | 0.307 | 0.307 | 0.307 | 0.307 |
| 2 | 1054.000 | 0.282 | 0.282 | 0.297 | 0.279 | 0.279 | 0.282 | 0.285 |
| 3 | 192.000 | 0.267 | 0.268 | 0.246 | 0.255 | 0.255 | 0.270 | 0.264 |
