# Supplementary material for: A comparison of the beta‐geometric model with landmarking for dynamic prediction of time to pregnancy
Source: Biom J. 2019 Nov 18;62(1):175–90. doi: 10.1002/bimj.201900155 (PMC6973003; doi:10.1002/bimj.201900155)
Supplement: Supplementary file 2 — Supporting Information [file BIMJ-62-175-s001.zip › Code/tabP_7.html]

|  | 1 | 2 | 3 | 4 | 5 | 6 | 7 | 8 |
| --- | --- | --- | --- | --- | --- | --- | --- | --- |
| 1 | 6000.000 | 0.289 | 0.290 | 0.352 | 0.287 | 0.286 | 0.289 | 0.289 |
| 2 | 1082.000 | 0.132 | 0.132 | 0.134 | 0.149 | 0.140 | 0.133 | 0.133 |
| 3 | 235.000 | 0.078 | 0.077 | 0.075 | 0.100 | 0.093 | 0.078 | 0.079 |
