# Supplementary material for: A comparison of the beta‐geometric model with landmarking for dynamic prediction of time to pregnancy
Source: Biom J. 2019 Nov 18;62(1):175–90. doi: 10.1002/bimj.201900155 (PMC6973003; doi:10.1002/bimj.201900155)
Supplement: Supplementary file 2 — Supporting Information [file BIMJ-62-175-s001.zip › Code/tabP_9.html]

|  | 1 | 2 | 3 | 4 | 5 | 6 | 7 | 8 |
| --- | --- | --- | --- | --- | --- | --- | --- | --- |
| 1 | 6000.000 | 0.291 | 0.291 | 0.353 | 0.293 | 0.291 | 0.290 | 0.288 |
| 2 | 1088.000 | 0.167 | 0.167 | 0.170 | 0.184 | 0.166 | 0.167 | 0.166 |
| 3 | 229.000 | 0.115 | 0.114 | 0.108 | 0.136 | 0.117 | 0.115 | 0.114 |
