# Supplementary material for: A comparison of the beta‐geometric model with landmarking for dynamic prediction of time to pregnancy
Source: Biom J. 2019 Nov 18;62(1):175–90. doi: 10.1002/bimj.201900155 (PMC6973003; doi:10.1002/bimj.201900155)
Supplement: Supplementary file 2 — Supporting Information [file BIMJ-62-175-s001.zip › Code/tabRMSEP_1.html]

|  | 1 | 2 | 3 | 4 | 5 | 6 | 7 | 8 |
| --- | --- | --- | --- | --- | --- | --- | --- | --- |
| 1 | 6000 | 35.2 | 35.2 | 36.0 | 35.2 | 35.2 | 37.1 | 0 |
| 2 | 1021 | 21.3 | 21.2 | 21.3 | 21.4 | 21.3 | 21.5 | 0 |
| 3 | 225 | 15.4 | 14.8 | 14.8 | 14.9 | 14.8 | 14.9 | 0 |
