# Supplementary material for: A comparison of the beta‐geometric model with landmarking for dynamic prediction of time to pregnancy
Source: Biom J. 2019 Nov 18;62(1):175–90. doi: 10.1002/bimj.201900155 (PMC6973003; doi:10.1002/bimj.201900155)
Supplement: Supplementary file 2 — Supporting Information [file BIMJ-62-175-s001.zip › Code/tabRMSEP_2.html]

|  | 1 | 2 | 3 | 4 | 5 | 6 | 7 | 8 |
| --- | --- | --- | --- | --- | --- | --- | --- | --- |
| 1 | 6000 | 31.8 | 31.9 | 32.7 | 31.8 | 31.8 | 34.6 | 0 |
| 2 | 992 | 20.8 | 20.7 | 20.7 | 20.6 | 20.6 | 22.0 | 0 |
| 3 | 211 | 16.3 | 15.5 | 15.4 | 15.2 | 15.2 | 16.3 | 0 |
