# Supplementary material for: A comparison of the beta‐geometric model with landmarking for dynamic prediction of time to pregnancy
Source: Biom J. 2019 Nov 18;62(1):175–90. doi: 10.1002/bimj.201900155 (PMC6973003; doi:10.1002/bimj.201900155)
Supplement: Supplementary file 2 — Supporting Information [file BIMJ-62-175-s001.zip › Code/tabRMSEP_5.html]

|  | 1 | 2 | 3 | 4 | 5 | 6 | 7 | 8 |
| --- | --- | --- | --- | --- | --- | --- | --- | --- |
| 1 | 6000 | 22.6 | 22.6 | 23.7 | 22.5 | 22.6 | 25.4 | 0 |
| 2 | 1031 | 23.4 | 23.3 | 23.4 | 23.7 | 23.4 | 24.0 | 0 |
| 3 | 202 | 21.8 | 21.6 | 21.6 | 23.1 | 22.1 | 21.2 | 0 |
