# Supplementary material for: A comparison of the beta‐geometric model with landmarking for dynamic prediction of time to pregnancy
Source: Biom J. 2019 Nov 18;62(1):175–90. doi: 10.1002/bimj.201900155 (PMC6973003; doi:10.1002/bimj.201900155)
Supplement: Supplementary file 2 — Supporting Information [file BIMJ-62-175-s001.zip › Code/tabRMSE_1.html]

|  | 1 | 2 | 3 | 4 | 5 | 6 | 7 | 8 |
| --- | --- | --- | --- | --- | --- | --- | --- | --- |
| 1 | 6000 | 0.796 | 0.804 | 7.00 | 0.736 | 0.756 | 0.797 | 0.506 |
| 2 | 1021 | 1.30 | 1.31 | 1.53 | 2.16 | 1.23 | 1.27 | 0.823 |
| 3 | 225 | 2.61 | 2.63 | 2.21 | 2.29 | 1.60 | 2.68 | 1.09 |
