# Supplementary material for: A comparison of the beta‐geometric model with landmarking for dynamic prediction of time to pregnancy
Source: Biom J. 2019 Nov 18;62(1):175–90. doi: 10.1002/bimj.201900155 (PMC6973003; doi:10.1002/bimj.201900155)
Supplement: Supplementary file 2 — Supporting Information [file BIMJ-62-175-s001.zip › Code/tabRMSE_10.html]

|  | 1 | 2 | 3 | 4 | 5 | 6 | 7 | 8 |
| --- | --- | --- | --- | --- | --- | --- | --- | --- |
| 1 |  | 0.802 | 0.811 | 7.02 | 0.739 | 0.745 | 0.796 | 0.449 |
| 2 |  | 1.27 | 1.27 | 1.49 | 2.12 | 1.17 | 1.27 | 0.671 |
| 3 |  | 2.63 | 2.65 | 2.22 | 2.23 | 1.51 | 2.68 | 0.968 |
