# Supplementary material for: A comparison of the beta‐geometric model with landmarking for dynamic prediction of time to pregnancy
Source: Biom J. 2019 Nov 18;62(1):175–90. doi: 10.1002/bimj.201900155 (PMC6973003; doi:10.1002/bimj.201900155)
Supplement: Supplementary file 2 — Supporting Information [file BIMJ-62-175-s001.zip › Code/tabRMSE_2.html]

|  | 1 | 2 | 3 | 4 | 5 | 6 | 7 | 8 |
| --- | --- | --- | --- | --- | --- | --- | --- | --- |
| 1 | 6000 | 0.857 | 0.863 | 7.23 | 0.78 | 0.732 | 0.851 | 0.455 |
| 2 | 992 | 1.52 | 1.51 | 1.81 | 0.937 | 0.816 | 1.51 | 0.691 |
| 3 | 211 | 2.64 | 2.71 | 2.39 | 0.793 | 0.73 | 2.63 | 1.10 |
