# Supplementary material for: A comparison of the beta‐geometric model with landmarking for dynamic prediction of time to pregnancy
Source: Biom J. 2019 Nov 18;62(1):175–90. doi: 10.1002/bimj.201900155 (PMC6973003; doi:10.1002/bimj.201900155)
Supplement: Supplementary file 2 — Supporting Information [file BIMJ-62-175-s001.zip › Code/tabRMSE_3.html]

|  | 1 | 2 | 3 | 4 | 5 | 6 | 7 | 8 |
| --- | --- | --- | --- | --- | --- | --- | --- | --- |
| 1 | 6000 | 0.853 | 0.86 | 6.91 | 0.703 | 0.743 | 0.86 | 0.503 |
| 2 | 1026 | 1.38 | 1.38 | 1.68 | 2.38 | 1.53 | 1.38 | 0.644 |
| 3 | 228 | 2.32 | 2.34 | 2.15 | 2.73 | 2.12 | 2.32 | 0.971 |
