# Supplementary material for: A comparison of the beta‐geometric model with landmarking for dynamic prediction of time to pregnancy
Source: Biom J. 2019 Nov 18;62(1):175–90. doi: 10.1002/bimj.201900155 (PMC6973003; doi:10.1002/bimj.201900155)
Supplement: Supplementary file 2 — Supporting Information [file BIMJ-62-175-s001.zip › Code/tabRMSE_4.html]

|  | 1 | 2 | 3 | 4 | 5 | 6 | 7 | 8 |
| --- | --- | --- | --- | --- | --- | --- | --- | --- |
| 1 | 6000 | 0.603 | 0.64 | 2.16 | 0.515 | 0.81 | 0.609 | 0.473 |
| 2 | 4033 | 0.517 | 0.515 | 0.527 | 2.24 | 0.277 | 0.508 | 0.33 |
| 3 | 3536 | 0.369 | 0.368 | 0.398 | 2.28 | 0.684 | 0.37 | 0.215 |
