# Supplementary material for: A comparison of the beta‐geometric model with landmarking for dynamic prediction of time to pregnancy
Source: Biom J. 2019 Nov 18;62(1):175–90. doi: 10.1002/bimj.201900155 (PMC6973003; doi:10.1002/bimj.201900155)
Supplement: Supplementary file 2 — Supporting Information [file BIMJ-62-175-s001.zip › Code/tabRMSE_5.html]

|  | 1 | 2 | 3 | 4 | 5 | 6 | 7 | 8 |
| --- | --- | --- | --- | --- | --- | --- | --- | --- |
| 1 | 6000 | 0.936 | 0.98 | 7.17 | 0.757 | 0.731 | 0.959 | 0.293 |
| 2 | 1031 | 1.62 | 1.61 | 2.11 | 3.08 | 1.37 | 1.63 | 0.617 |
| 3 | 202 | 3.49 | 3.38 | 3.22 | 6.46 | 3.56 | 3.56 | 1.49 |
