# Supplementary material for: A comparison of the beta‐geometric model with landmarking for dynamic prediction of time to pregnancy
Source: Biom J. 2019 Nov 18;62(1):175–90. doi: 10.1002/bimj.201900155 (PMC6973003; doi:10.1002/bimj.201900155)
Supplement: Supplementary file 2 — Supporting Information [file BIMJ-62-175-s001.zip › Code/tabRMSE_6.html]

|  | 1 | 2 | 3 | 4 | 5 | 6 | 7 | 8 |
| --- | --- | --- | --- | --- | --- | --- | --- | --- |
| 1 | 6000 | 0.777 | 0.781 | 6.29 | 0.727 | 0.727 | 0.806 | 0.128 |
| 2 | 1054 | 2.14 | 2.14 | 2.81 | 1.14 | 1.17 | 2.22 | 0.382 |
| 3 | 192 | 4.80 | 4.73 | 4.51 | 1.93 | 1.95 | 4.84 | 0.896 |
