# Supplementary material for: A comparison of the beta‐geometric model with landmarking for dynamic prediction of time to pregnancy
Source: Biom J. 2019 Nov 18;62(1):175–90. doi: 10.1002/bimj.201900155 (PMC6973003; doi:10.1002/bimj.201900155)
Supplement: Supplementary file 2 — Supporting Information [file BIMJ-62-175-s001.zip › Code/tabRMSE_7.html]

|  | 1 | 2 | 3 | 4 | 5 | 6 | 7 | 8 |
| --- | --- | --- | --- | --- | --- | --- | --- | --- |
| 1 | 6000 | 0.787 | 0.787 | 6.42 | 0.722 | 0.722 | 0.799 | 0.447 |
| 2 | 1082 | 1.48 | 1.48 | 1.82 | 2.02 | 0.975 | 1.50 | 0.703 |
| 3 | 235 | 2.25 | 2.25 | 1.91 | 2.43 | 1.48 | 2.30 | 1.03 |
