# Supplementary material for: A comparison of the beta‐geometric model with landmarking for dynamic prediction of time to pregnancy
Source: Biom J. 2019 Nov 18;62(1):175–90. doi: 10.1002/bimj.201900155 (PMC6973003; doi:10.1002/bimj.201900155)
Supplement: Supplementary file 2 — Supporting Information [file BIMJ-62-175-s001.zip › Code/tabRMSE_8.html]

|  | 1 | 2 | 3 | 4 | 5 | 6 | 7 | 8 |
| --- | --- | --- | --- | --- | --- | --- | --- | --- |
| 1 | 6000 | 0.771 | 0.767 | 6.07 | 0.744 | 0.775 | 0.761 | 0.442 |
| 2 | 1092 | 1.57 | 1.56 | 1.82 | 1.67 | 1.13 | 1.60 | 0.578 |
| 3 | 239 | 2.21 | 2.15 | 1.88 | 2.18 | 1.73 | 2.14 | 0.771 |
