# Supplementary material for: A comparison of the beta‐geometric model with landmarking for dynamic prediction of time to pregnancy
Source: Biom J. 2019 Nov 18;62(1):175–90. doi: 10.1002/bimj.201900155 (PMC6973003; doi:10.1002/bimj.201900155)
Supplement: Supplementary file 2 — Supporting Information [file BIMJ-62-175-s001.zip › Code/tabRMSE_9.html]

|  | 1 | 2 | 3 | 4 | 5 | 6 | 7 | 8 |
| --- | --- | --- | --- | --- | --- | --- | --- | --- |
| 1 | 6000 | 0.88 | 0.906 | 6.62 | 0.854 | 0.76 | 0.865 | 0.369 |
| 2 | 1088 | 1.42 | 1.40 | 1.79 | 2.67 | 0.936 | 1.48 | 0.651 |
| 3 | 229 | 3.06 | 3.01 | 2.67 | 3.08 | 0.979 | 3.04 | 0.94 |
