# Supplementary figures and images for: A comparison of the beta‐geometric model with landmarking for dynamic prediction of time to pregnancy
Source: Biom J. 2019 Nov 18;62(1):175–90. doi: 10.1002/bimj.201900155 (PMC6973003; doi:10.1002/bimj.201900155)

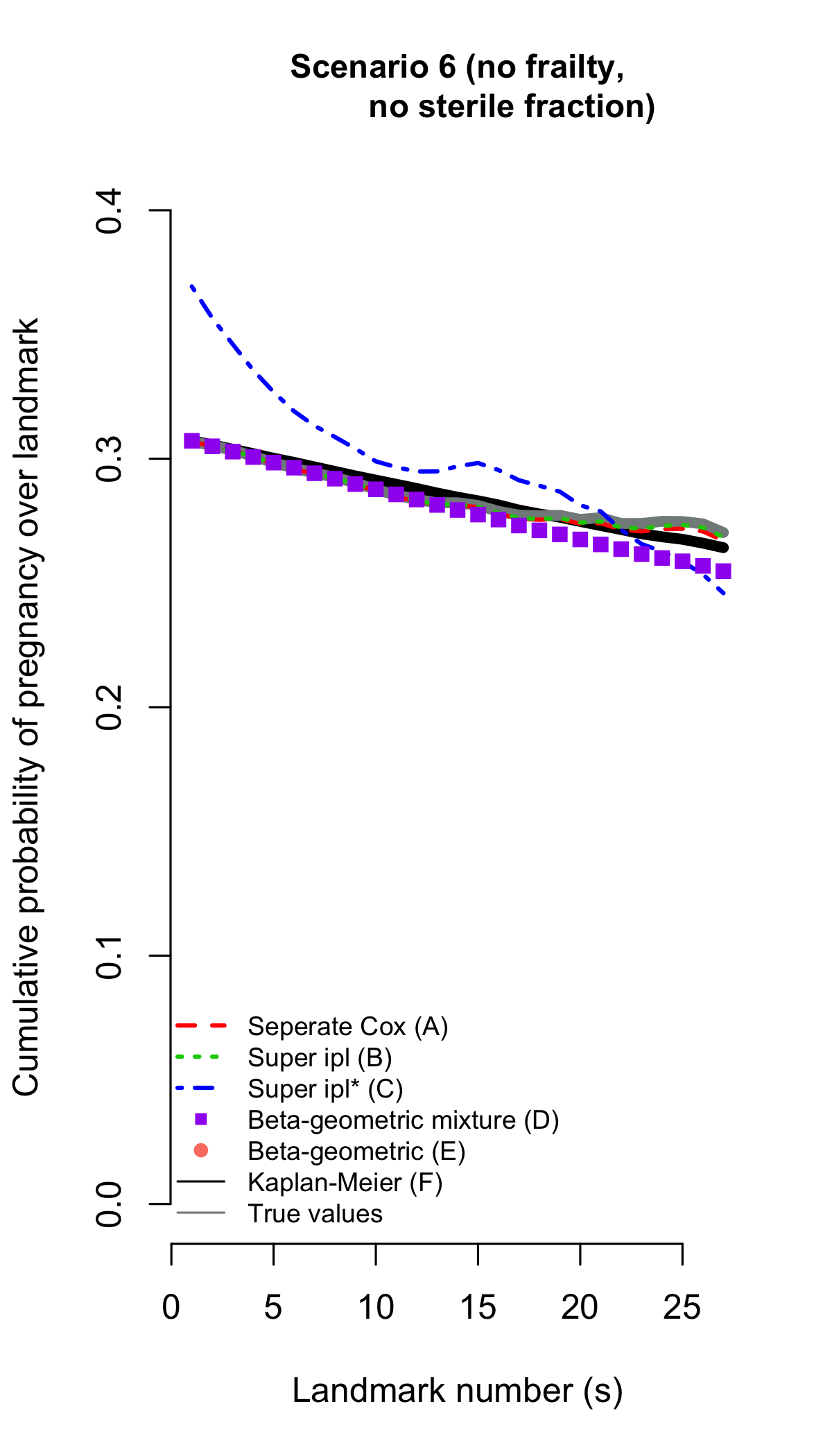

Supplement: Supplementary file 2 — Supporting Information [file BIMJ-62-175-s001.zip › Code/res_0_nosteril_pred.tiff]

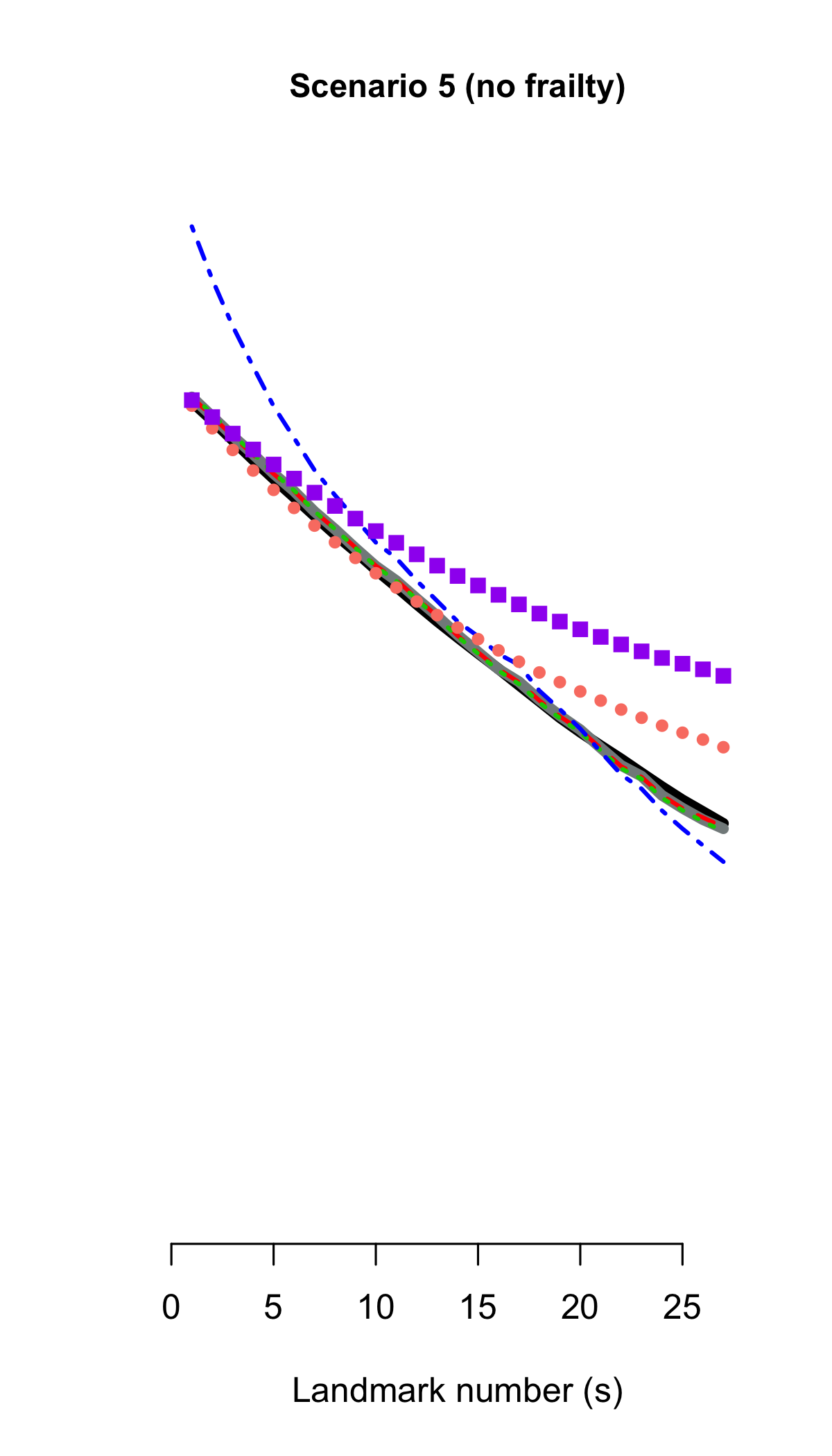

Supplement: Supplementary file 2 — Supporting Information [file BIMJ-62-175-s001.zip › Code/res_0_pred.tiff]

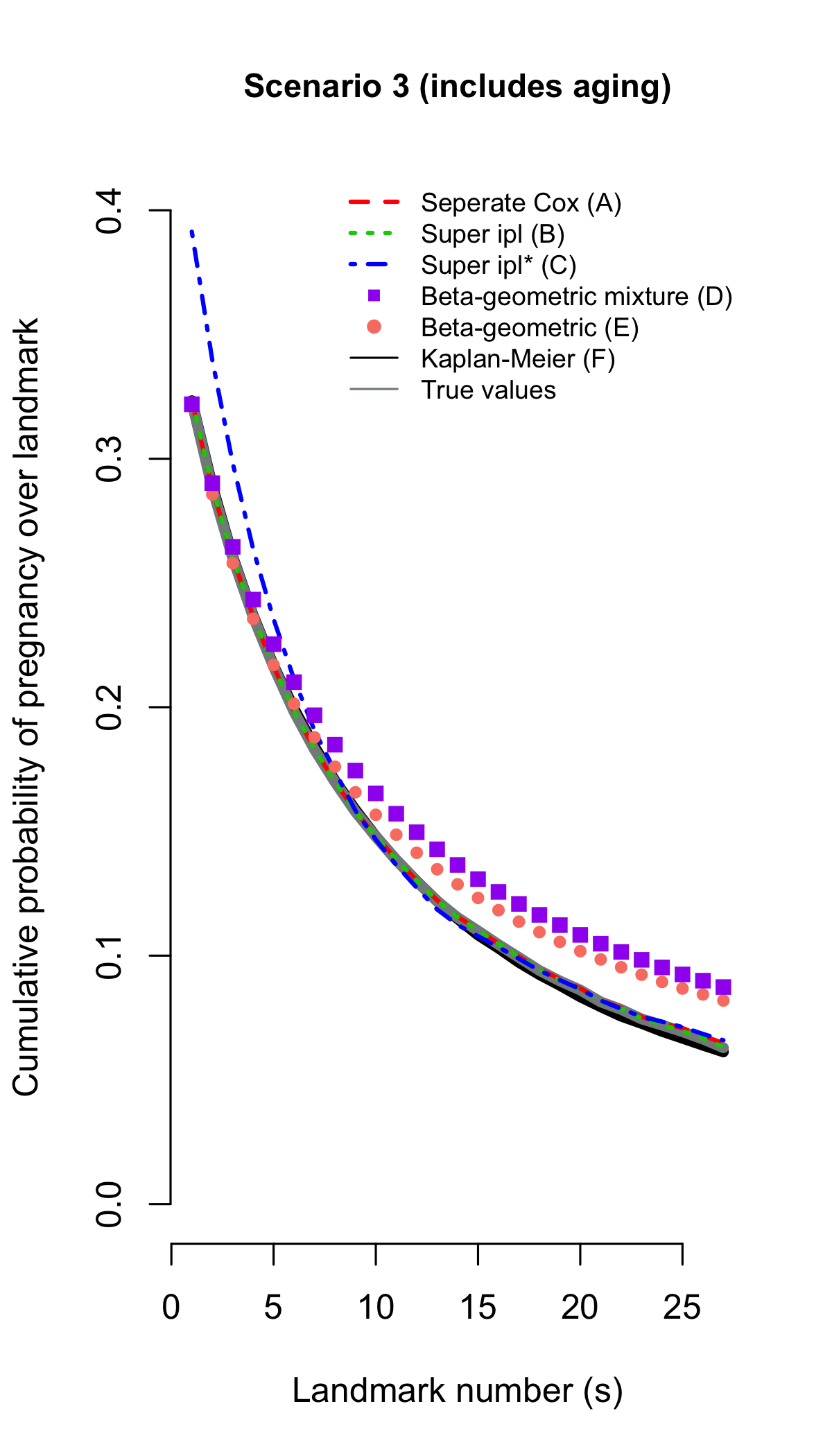

Supplement: Supplementary file 2 — Supporting Information [file BIMJ-62-175-s001.zip › Code/res_bg_aging_pred.tiff]

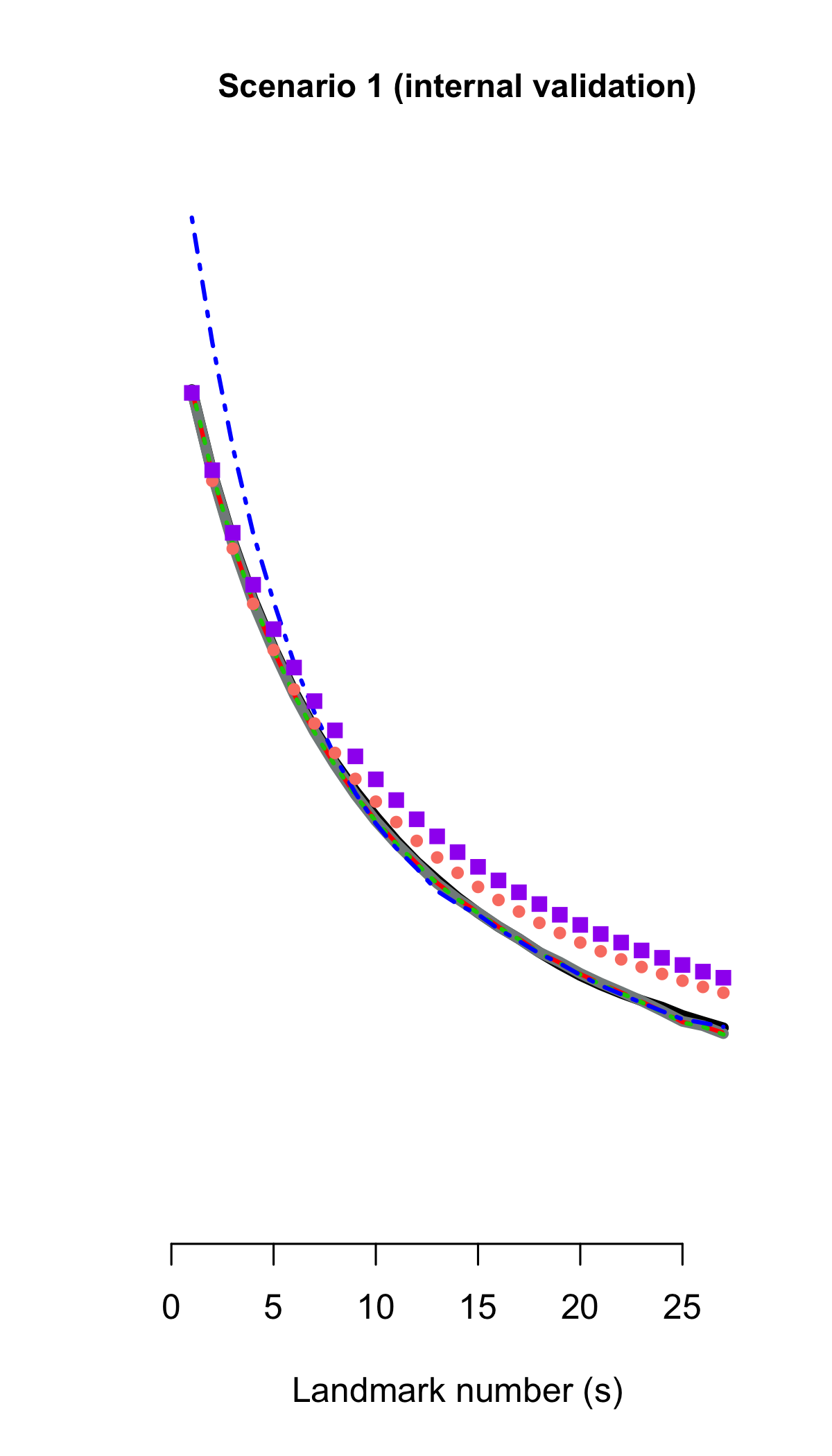

Supplement: Supplementary file 2 — Supporting Information [file BIMJ-62-175-s001.zip › Code/res_bg_intern_pred.tiff]

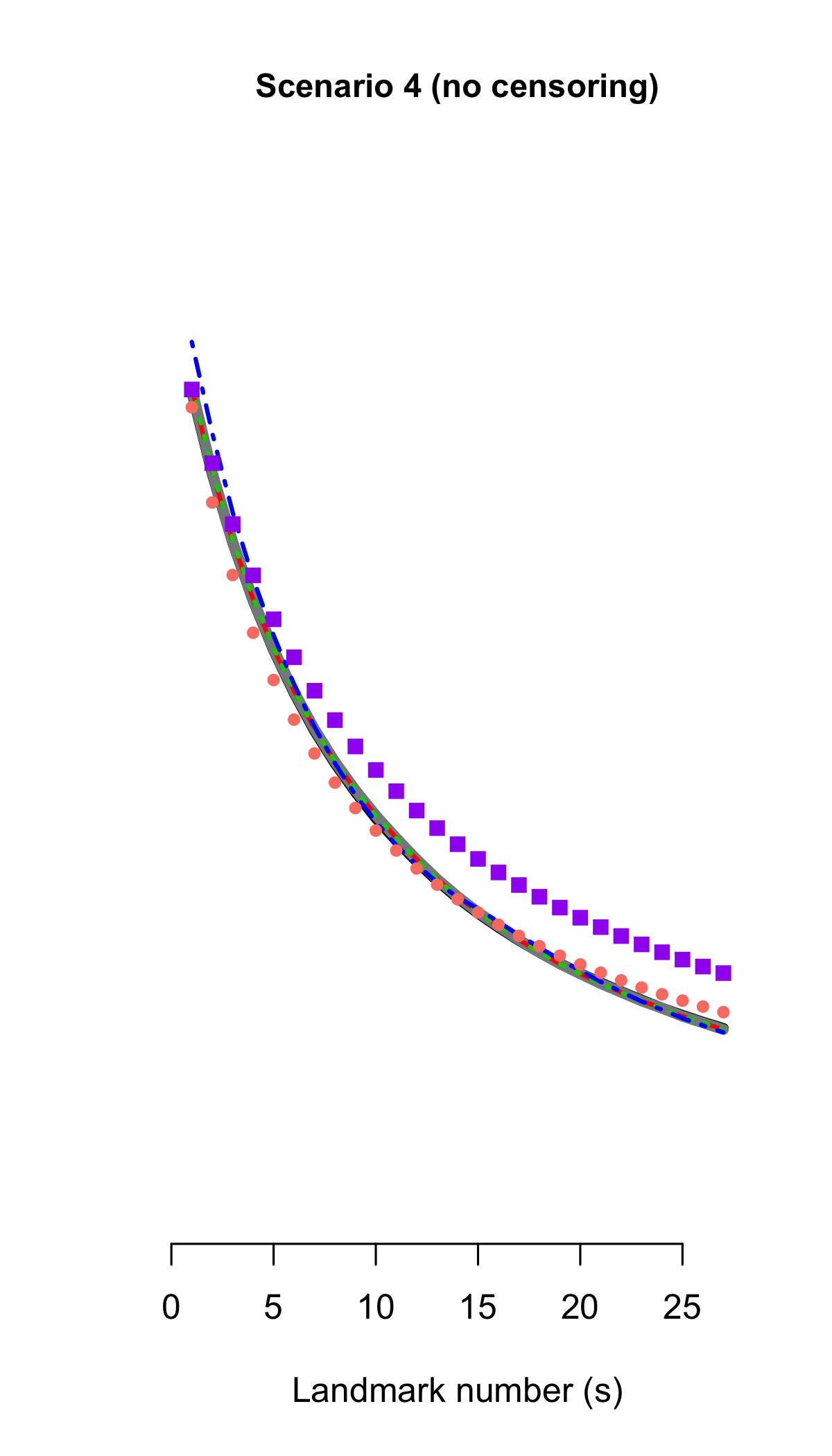

Supplement: Supplementary file 2 — Supporting Information [file BIMJ-62-175-s001.zip › Code/res_bg_nocens_pred.tiff]

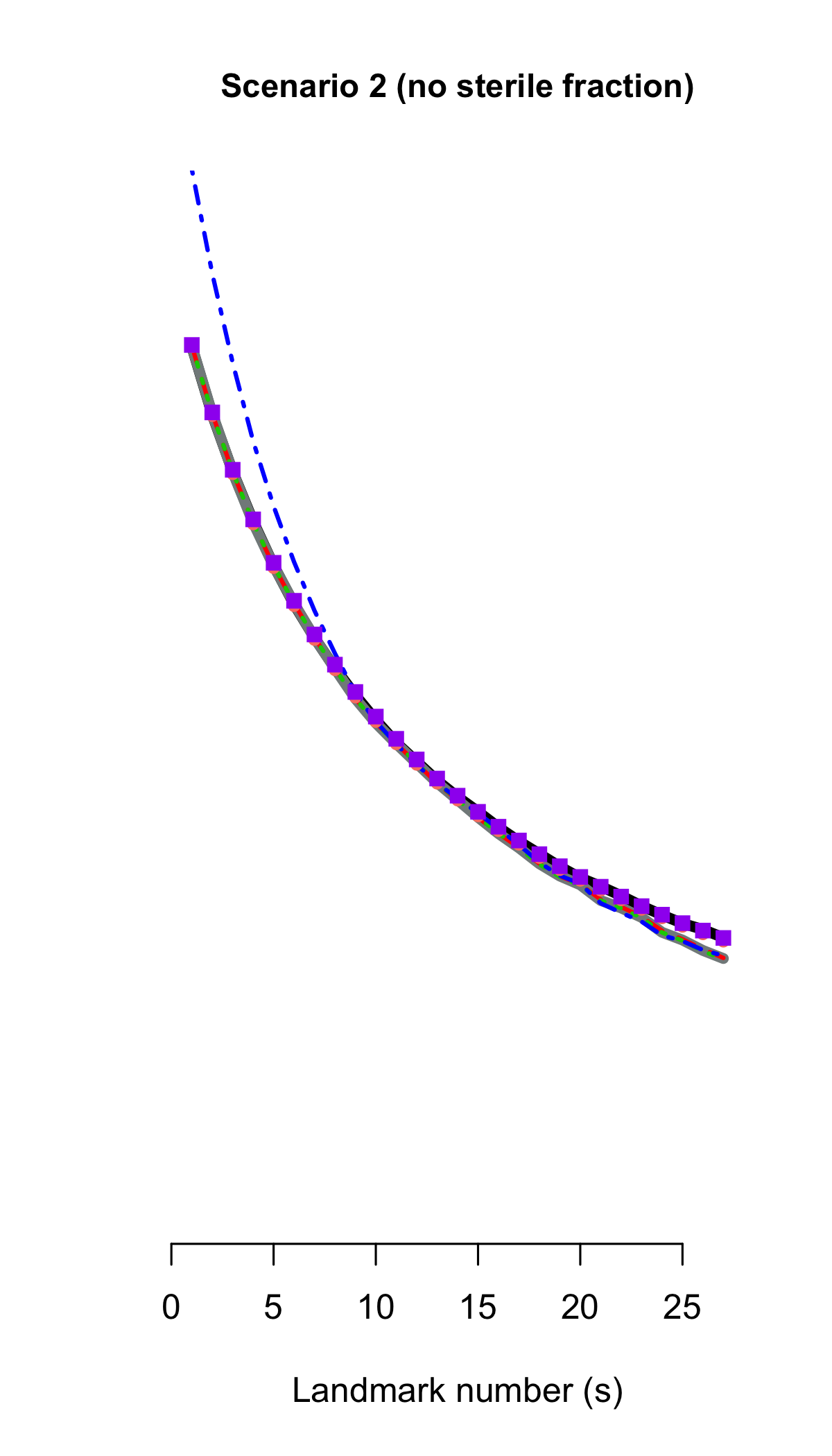

Supplement: Supplementary file 2 — Supporting Information [file BIMJ-62-175-s001.zip › Code/res_bg_nosteril_pred.tiff]

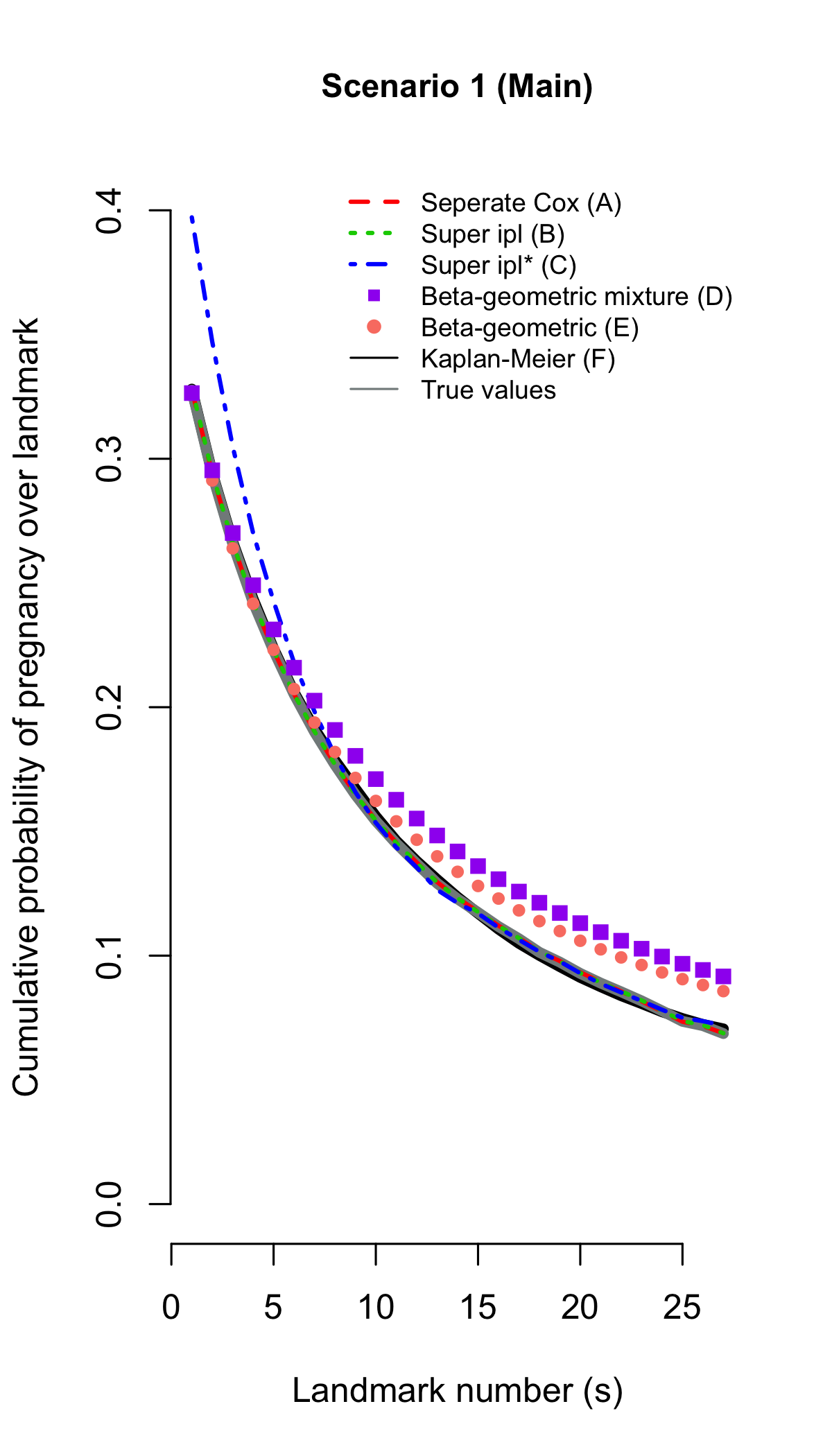

Supplement: Supplementary file 2 — Supporting Information [file BIMJ-62-175-s001.zip › Code/res_bg_pred.tiff]

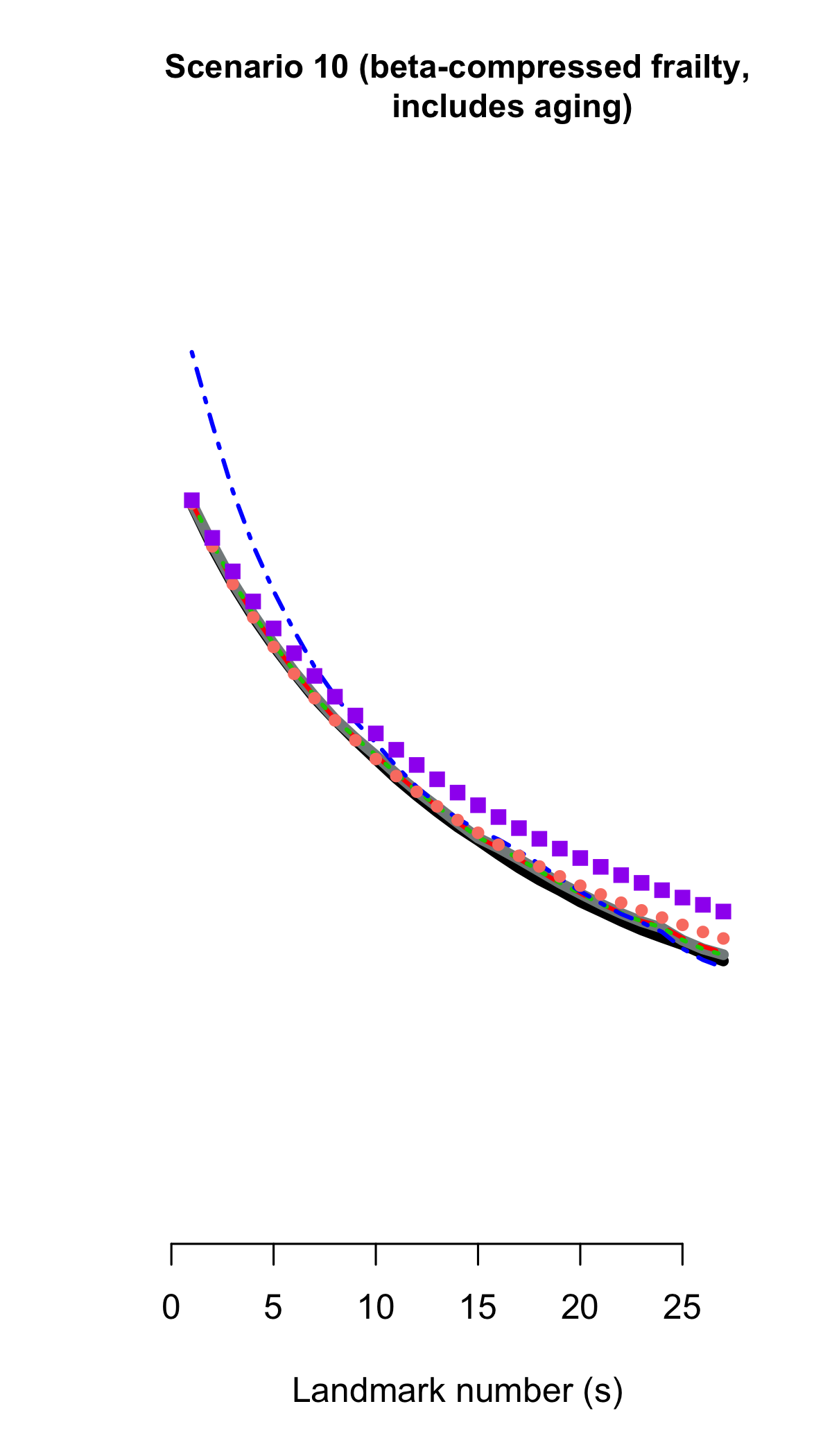

Supplement: Supplementary file 2 — Supporting Information [file BIMJ-62-175-s001.zip › Code/res_compr_aging_pred.tiff]

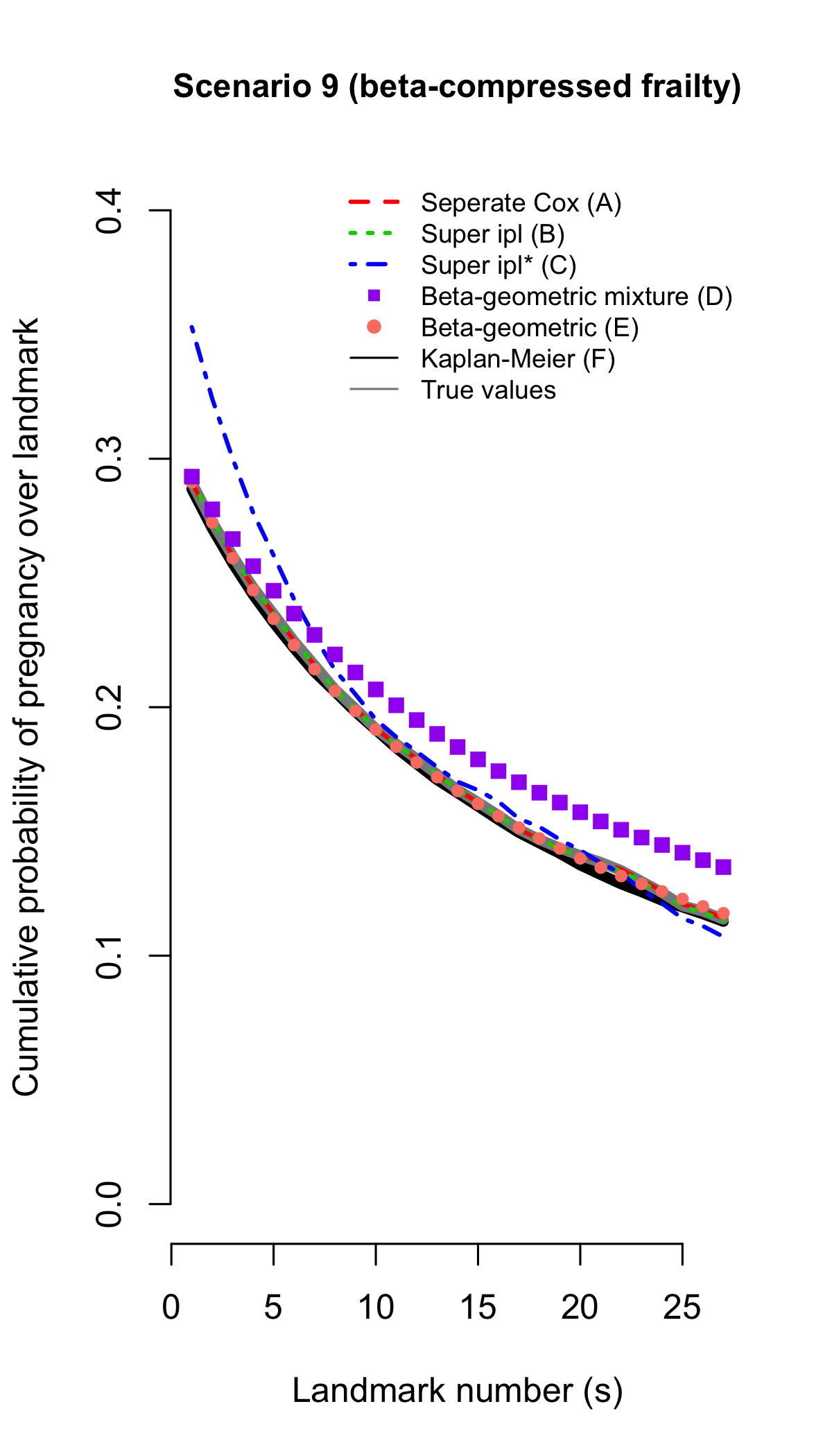

Supplement: Supplementary file 2 — Supporting Information [file BIMJ-62-175-s001.zip › Code/res_compr_pred.tiff]

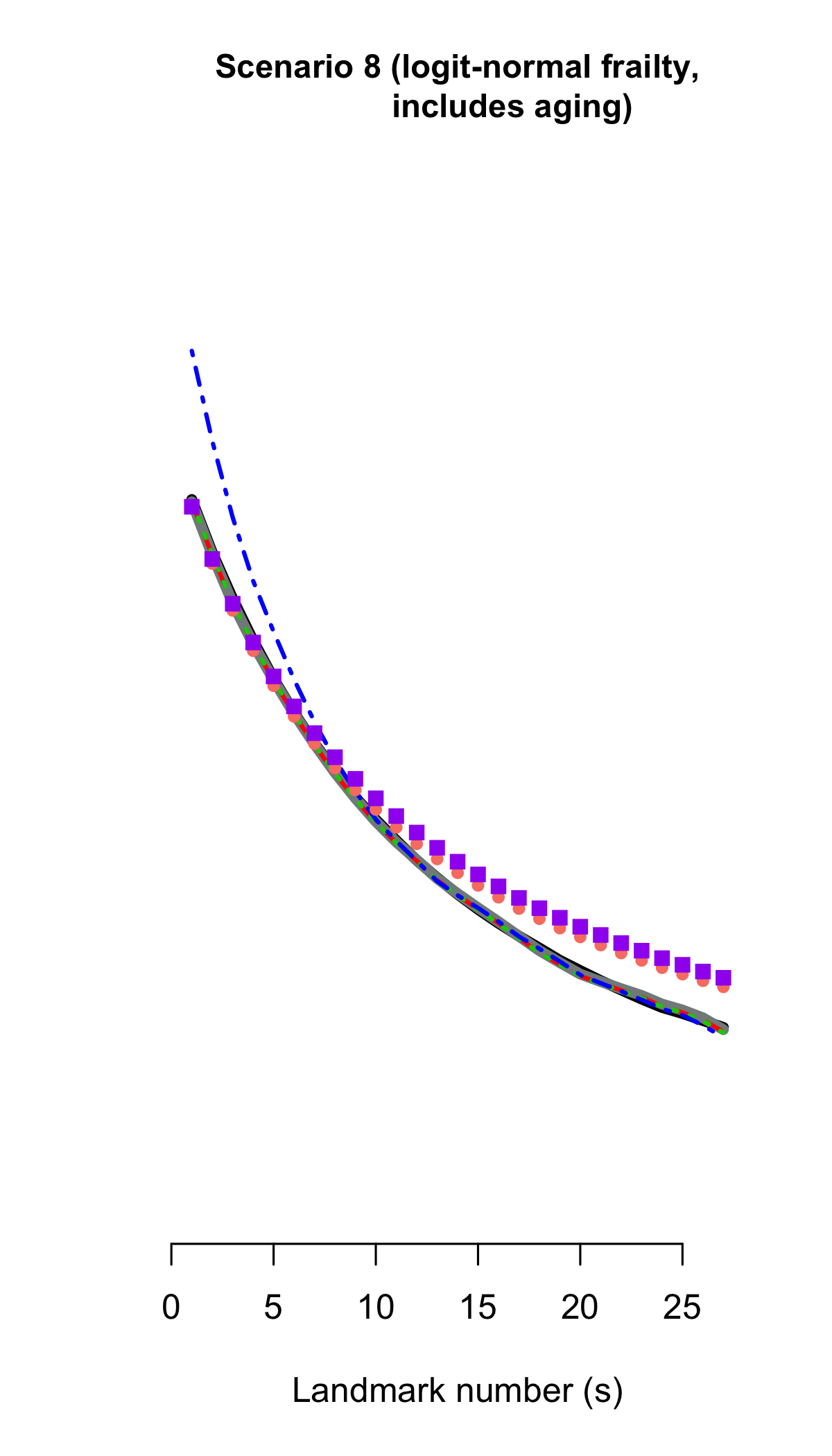

Supplement: Supplementary file 2 — Supporting Information [file BIMJ-62-175-s001.zip › Code/res_normal_aging_pred.tiff]

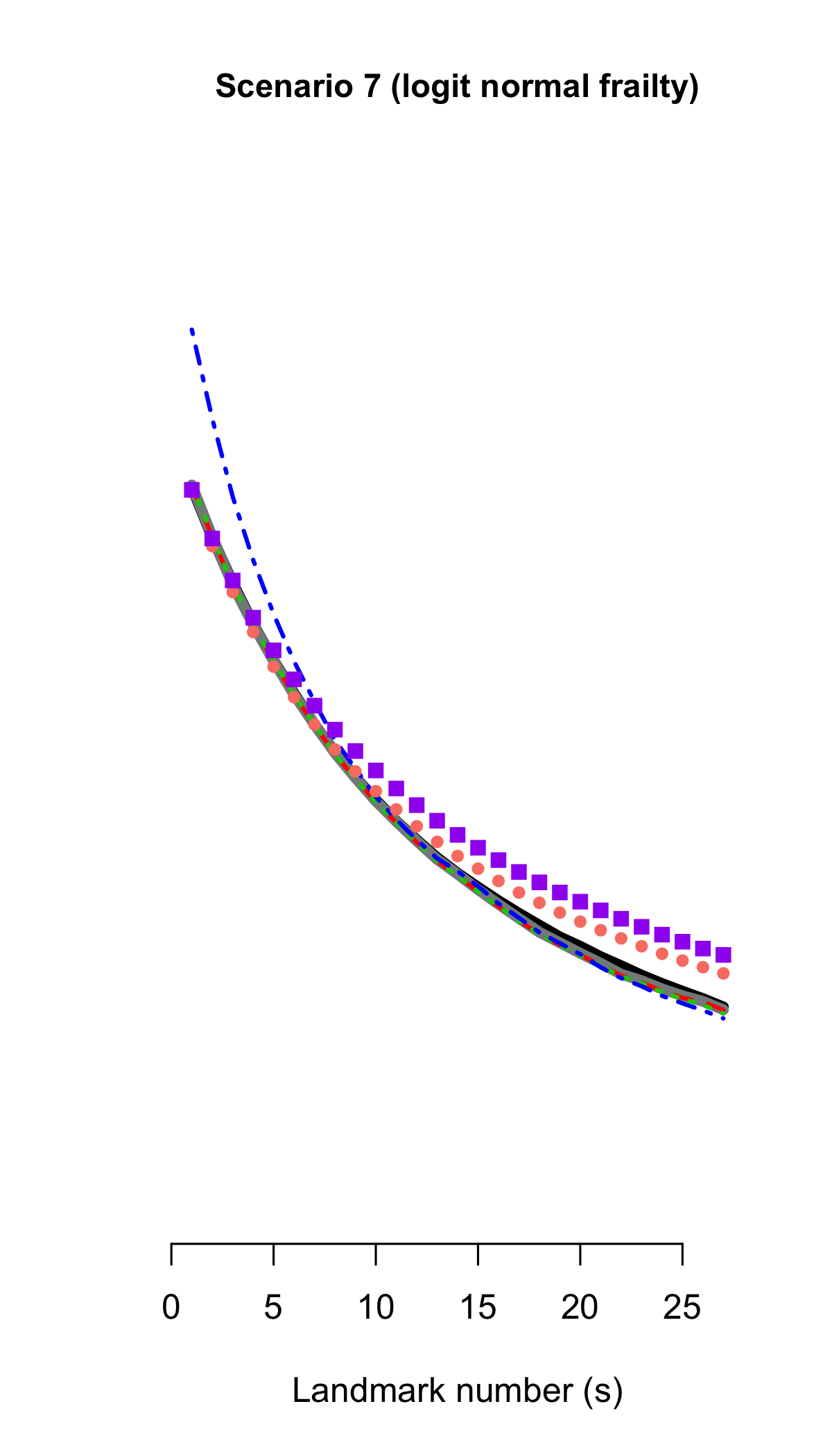

Supplement: Supplementary file 2 — Supporting Information [file BIMJ-62-175-s001.zip › Code/res_normal_pred.tiff]
